# Multi-Modal Multi-Spectral Intravital Microscopic Imaging of Signaling Dynamics in Real-Time during Tumor–Immune Interactions

**DOI:** 10.3390/cells10030499

**Published:** 2021-02-26

**Authors:** Tracy W. Liu, Seth T. Gammon, David Piwnica-Worms

**Affiliations:** Department of Cancer Systems Imaging, University of Texas MD Anderson Cancer Center, Houston, TX 77030, USA; twliu@mdanderson.org (T.W.L.); stgammon@mdanderson.org (S.T.G.)

**Keywords:** intravital microscopy, bioluminescence microscopy, immune cell imaging, imaging reporters, molecular imaging, tumor signaling, tumor–immune interactions

## Abstract

Intravital microscopic imaging (IVM) allows for the study of interactions between immune cells and tumor cells in a dynamic, physiologically relevant system in vivo. Current IVM strategies primarily use fluorescence imaging; however, with the advances in bioluminescence imaging and the development of new bioluminescent reporters with expanded emission spectra, the applications for bioluminescence are extending to single cell imaging. Herein, we describe a molecular imaging window chamber platform that uniquely combines both bioluminescent and fluorescent genetically encoded reporters, as well as exogenous reporters, providing a powerful multi-plex strategy to study molecular and cellular processes in real-time in intact living systems at single cell resolution all in one system. We demonstrate that our molecular imaging window chamber platform is capable of imaging signaling dynamics in real-time at cellular resolution during tumor progression. Importantly, we expand the utility of IVM by modifying an off-the-shelf commercial system with the addition of bioluminescence imaging achieved by the addition of a CCD camera and demonstrate high quality imaging within the reaches of any biology laboratory.

## 1. Introduction

Intravital microscopic imaging (IVM) has proven to be a powerful imaging technique [1,2,3,4,5,6,7,8,9,10,11], one of the few molecular imaging strategies that provides both spatial and temporal information regarding heterogeneous living systems in real-time at cellular resolution. In particular, this technology has become increasingly critical in studies of the tumor immune microenvironment, as it has become increasingly recognized that the immune system plays complex dual roles in cancer, both beneficially and adversely impacting tumorigenesis [12,13,14,15]. Thus, advances that allow for the study of interactions between immune cells and tumor cells in a dynamic, physiologically relevant system in vivo have become crucial. Enormous advances in immunology have been achieved though the analysis of tissues in bulk at different stages of tumor progression as well as a variety of in vitro and ex vivo methodologies. However, many of these findings provide only a snapshot in time of orchestrated molecular and cellular changes occurring over time. Analysis of tissue in bulk (e.g., Western blots, whole genome sequencing, PCR, RPPA) disregards spatial and temporal distributions and relationships during disease development and ignores the often massive heterogeneity of tumor cells themselves (genotype and phenotype), as well as heterogeneity within the immune cell microenvironment. Advances in single cell digital techniques (single cell RNAseq, CyTOF and multiplex imaging) have laid the foundational framework for advances in cancer immunology that account for cellular heterogeneity; however, these techniques are destructive and time-course dynamics require a large number of subjects for analysis of genetic, molecular and cellular changes that cannot be tracked or sampled temporally or spatially within the same individual. Only through non-invasive imaging can tumor progression and dynamic immune cell interaction in an intact in vivo system be monitored in real-time. However, few imaging modalities can achieve non-invasive cellular resolution sufficient to image the molecular mechanisms of cell trafficking, cell–cell interactions and the associated molecular signals that IVM provides.

Current IVM strategies primarily use fluorescence imaging [1,2,3,4,5,6,7]. Unfortunately, fluorescence has several limitations, including high background (autofluorescence) that renders the quantification of fluorescence difficult, resulting in low signal-to-noise ratios, photo-bleaching, and expensive imaging systems [1,2,3,4,5,6,7]. Although there are numerous fluorescent proteins and/or exogenous fluorochromes, current IVM microscopy systems are generally limited in the number of fluorophores that can be imaged simultaneously [1,2,3,4,5,6,7]. In addition, many fluorescent proteins have long half-lives, e.g., green fluorescent protein (GFP) has a half-life of ~12–26 h [16,17,18], which limits the capacity to quantify on–off dynamics in biological systems. In contrast, bioluminescence has become a standard preclinical macro-imaging tool for monitoring cancer cell fate and tumor growth [11,16,17,18,19]. Traditionally, tumor cells are stably engineered to express constitutively active bioluminescent reporters, which directly measure live cell metabolism [11,16,17,19,20,21,22]. Bioluminescence has several advantages over fluorescence, including low background signal, high signal-to-noise ratios, and modest cost [23,24,25]. In addition, luciferases have a short half-life, e.g., North American *Photinus pyralis* firefly and *Renilla* luciferase have a half-life of ~ 3–5 h, where their rapid translation and maturation better reflects endogenous biological activation on-rates [17]. With the development of new bioluminescent reporters with expanded emission spectra, advances in genomic techniques, and technical improvements in bioluminescence imaging and processing techniques [11,16,17,18,19], the applications for bioluminescence imaging are increasing. Recent advances in microscopy technologies are extending bioluminescence applications to single cell imaging [26,27,28,29,30].

A limitation of bioluminescence is the substrate dependence of luciferase enzymes where the pharmacokinetics of substrate delivery temporally impacts the bioluminescence signal in vivo. Unlike fluorophores, there are a limited number of bioluminescent proteins, although three luciferases have been spectrally unmixed macroscopically [31,32]. Luciferases, such as firefly luciferase and click beetle green luciferase, which both use the same D-luciferin substrate, can be imaged, and discriminated simultaneously [11,16,17,19,31,33,34,35]. Multi-color bioluminescence can be separated using appropriate emission filters and de-convoluted using spectral unmixing algorithms [31,32]. In addition, bioluminescent reporters that utilize different substrates are easily resolved in the same animal with separate sequential imaging sessions [11,16,17,19,31,32,33,34,35]. Bioluminescent reporters have been developed that can image biological events at multiple levels; monitoring the regulation of specific genes, messenger RNA processing, signal transduction, protein processing and function, and protein–protein interactions are possible using transcriptional, translational and post-translational bioluminescent reporters [11,16,17,18,19]. The combination of various reporters (genetic, protein and/or cellular) with different luciferases provides a powerful approach to study the temporal and spatial evolution of biological processes in vivo, wherein different molecular and/or cellular events can be monitored by bioluminescence simultaneously in a single imaging session. Herein, we describe a molecular imaging window chamber platform that uniquely combines bioluminescent and fluorescent reporters with intravital microscopy, providing a first-in-class advance in technology of intravital spectral unmixing in real-time concurrent with high resolution bioluminescence imaging of signaling dynamics applicable to heterogeneous living systems in vivo.

## 2. Materials and Methods

### 2.1. Microscope Setup and Configuration

A Nikon TiE inverted microscope (Nikon Instruments, Melville, NY, USA) provided the core scaffold for the custom imaging system and was used for all intravital imaging studies. The microscope was equipped with the following objectives: 2X NA 0.1 8.5 mm WD (CFI Plan Apochromat Lambda 2X), 10X NA 0.45 (Plan Apo) and 20X NA 0.45 (s Plan Fluor extra-long working distance) (Nikon Instruments, Melville, NY, USA). The internal reflection of NIR light from the real time focal adjustment system (Perfect Focus, Nikon Instruments) was blocked by placing a 25 mm 750 nm short pass filter with >6 OD stopping power and >90% transmittance (ET750sp-2p8, Chroma Technology Corp, Bellows Falls, VT, USA) in the filter wheel (FLBW-E, Nikon Instrument) either alone or in series with 25 mm short pass, band pass, and long pass filters (BrightLine FF01-492/SP-25, Brightline FF01-540/50, Edgebasic BLP01-635R, Semrock Corporation Rochester, NY, USA). An open slot was maintained for use during fluorescence imaging (including NIR). The filter wheel was placed directly in line with the camera system, enabling both the collection of single reporter bioluminescence imaging (750 nm short pass), or multispectral bioluminescence imaging (750 nm short pass in series with the appropriate visible filter). This filter wheel was under the automated control of the microscope for the facile programming of single acquisition and multiple mixed fluorescence and bioluminescence protocols. For bioluminescence and epifluorescence imaging, a back-illuminated 1024 × 1024 pixel CCD with a 13 µm pixel pitch (iKon-M 934; DU934P-BEX2-DD, Andor Inc/Oxford Instruments Belfast, Northern Ireland) was air cooled to −85 °C during normal operations with deep depletion fringe suppression and anti-reflective coating. When acquiring bioluminescence images, the camera was read out at 50 kHz, 4X gain, and binning 2 to minimize read noise and maximize sensitivity. In the epifluorescence mode, the readout speed was 1 MHz. Confocal imaging was integrated into the platform using the LSM C2 Confocal system (Nikon Instruments, Melville, NY, USA). The isoflurane vaporizer and induction chamber were placed on a separate table adjacent to the microscope table to minimize vibrations during imaging. An isoflurane vaporizer (XGI-8, Perkin Elmer, Waltham, MA, USA) supplied isoflurane and air plus a vacuum to anaesthetize animals. The induction chamber was placed onto a custom 24′′ × 24′′ perforated downdraft table (Biomedical Solutions Inc, Stafford, TX, USA) to further minimize isoflurane exposure to the operator. Furthermore, a continuous low flow “snorkel” was placed near the nose cone on the stage to further minimize exposure of isoflurane. A single beam infrared spectrophotometer (MIRAN SapphIRe gas analyzer, Thermo Environmental Instruments, Franklin, MA, USA) was utilized to measure the instantaneous isoflurane exposure rates at the microscope, near the nose cone, and at the isoflurane vaporizer, induction box, induction box connectors, filters, and breathing zone above the mouse. A custom heated stage top (Oko Lab, NA, Italy) was designed for intravital imaging with a stage heating system (h401-t-single-bl, Oko Lab, NA, Italy) to ensure animals were maintained at 37 °C during image acquisition.

### 2.2. iKon Camera Characterization

The camera was tested to independently validate the read noise both globally and on a per pixel basis. In total, 300 short (100 µsec) images were acquired with the camera shutter closed. A bias image was generated by calculating the median value at each pixel to reject the effects of cosmic ray strikes. Both a median absolute deviation image and a 5-sigma filtered standard deviation image were generated. Read noise was calculated via 5-sigma filtering of all pixels in all 300 images, and then calculating the global standard deviation. Read noise was well controlled (3 Gl), with very small but detectable top to bottom and left to right gradients in read noise: 4.9 × 10^−4^ Gl/pixel and 1.8 × 10^−6^ Gl/pixel, respectively. The number of electrons was calculated from vendor-supplied electron-Gl conversion values for a 50 kHz read and 4X gain (1.2) and the number of read photons required by accounting for 0.95 quantum yield. Test–retest data and correlation with a macroscopic imaging system (IVIS Spectrum) were conducted with an NIST traceable luminometer microplate reference standard (LRM 168-96 622 nm emission, HARTA Instruments Gaithersburg, MD, USA).

### 2.3. Reagents 

D-Luciferin, potassium salt, was purchased from Gold Biotechnology, Inc.^®^ (St. Louis, MO, USA). TNFα was purchased from R&D Systems, Inc. (Minneapolis, MN, USA). L-012 sodium salt was purchased from Wako Chemicals USA (Richmond, VA, USA). L-012 powder was dissolved in sterile double distilled water (ddH_2_0) to a final concentration of 20 mM and stored at −20 °C. Puromycin was purchased from Thermo Fisher Scientific (Waltham, MA, USA).

### 2.4. Cells 

B16F10 melanoma cells were purchased from the MD Anderson Cancer Center Cell core (originally from the American Type Culture Collection, ATCC, Manassas, VA, USA). B16F10 cells were cultured in DMEM supplemented with 10% heat-inactivated fetal bovine serum (FBS). Pan02 cells were purchased from the DCTD Tumor Repository, National Cancer Institute (Frederick, Maryland). Pan02 cells were cultured in RPMI 1640 supplemented with 10% heat-inactivated FBS and 2 mM glutamine. Cell cultures were grown at 37 °C in a humidified 5% CO_2_ atmosphere.

### 2.5. Plasmids

The κB5→IκBα-FLuc and κB5→FLuc plasmid were previously described [30,36]. The FUW-FLG plasmid encoding a fusion of CBG and EGFP proteins driven by the human ubiquitin C promoter within an established lentiviral backbone has been previously described [37]. The pFLuc control plasmid was generated as previously described [25]. The GFP-IRES-GFP lentivirus was a gift from Ron DePinho (UT MD Anderson Cancer Center, TX, USA). The pDendra2-N and pLVX-IRES-Puro were purchased from Clontech (Clontech, Moutain View, CA, USA). A Cignal lenti NFκB→FLuc reporter was purchased from Qiagen (SABiosceinces, Frederick, MD, USA).

### 2.6. Generation of Stable Reporter Cells

The generation of a B16F10 cell line stably expressing GFP: B16F10 cells at 50% confluence were infected with the second generation GFP-IRES-GFP, CBG or FLuc lentiviruses. GFP-IRES-GFP and CBG infected B16F10 cells were selected for GFP expression by FACS sorting. FLuc infected B16F10 cells were selected by bioluminescence imaging; after two additional weeks, bioluminescence images of isolated cell colonies using phenol free DMEM media with 10% FBS and 150 µg/mL D-Luciferin were acquired to assess reporter gene expression, upon which bioluminescent colonies were harvested and expanded. Cells were transduced at a multiplicity of infection (MOI) of 10. The generation of the B16F10 GFP-IRES-GFP cell line stably expressing pκB5→FLuc: cells at 95% confluence was co-transfected with 10 µg of pκB5→FLuc plasmid DNA and 3 µg of pLVX-IRES-Puro plasmid DNA using Fugene6 transfection reagent (Promega, Madison, WI, USA) in a 6-well plate. At 24 h post transfection, media were replaced with fresh cell media, and at 48 h post transfection, cells were trypsinized and plated at a variety of densities (1:2, 1:5, 1:10) into media containing 2 µg/mL puromycin to select for stable transformants. After two weeks, bioluminescence images of isolated cell colonies using phenol free DMEM media with 10% FBS and 150 µg/mL D-Luciferin were acquired to assess reporter gene expression, upon which bioluminescent colonies were harvested and expanded. Cells were continuously cultured in the presence of 2 µg/mL puromycin to maintain expression of the reporter plasmid. The generation of a B16F10 and Pan02 cell line stably expressing Dendra2: cells at 95% confluence were co-transfected with 10 µg of pDendra2-N plasmid DNA using Fugene6 in a 6-well plate. At 24 h post transfection, media were replaced with fresh cell media, and at 48 h post transfection, cells were trypsinized and plated in media containing 1.5 mg/mL G418 for B16F10 cells and 150 µg/mL G418 for Pan02 cells to select for stable transformants. Flow cytometry was used to isolate Dendra2-positive cells; cells were sorted twice for Dendra2 fluorescence (λ_ex_ = 409 nm, λ_em_ = 507 nm). The generation of a B16F10 Dendra2 and Pan02 Dendra2 cell stably expressing NF-κB→FLuc: B16F10 Dendra2 or Pan02 Dendra2 cells at 50% confluence were transduced with the NF-κB→FLuc lentivirus and selected using 2 µg/mL puromycin. Cells were transduced at a MOI of 10. All cell lines tested negative for mycoplasma.

### 2.7. Animals

The Institutional Animal Care and Use Committee at the University of Texas MD Anderson Cancer Center approve all animal protocols. The following animals were used for window chamber experiments: wild type C57BL/6 animals (Taconic Biosciences, Rensselaer, NY, USA); syngeneic C57BL/6 myeloperoxidase-deficient *MPO^−/−^* (*MPO^tm^*^1*Lus*^, The Jackson Laboratory, Bar Harbor, ME, USA) mice; *p*21-*FLuc* reporter mice [26]; and *LSL-Kras^G12D/+^;LSL-p53^T172H/+^;Pdx-1-Cre* (*KPC*) animals were kindly provided by Dr. Chun Li [38].

### 2.8. Preparation for Window Chamber Implantation Surgery

Animals weighed at least 20 g prior to window chamber implantation surgery. Surgical kits with chlorhexidine, ophthalmic ointment, buprenorphine and sterile materials were provided from the Department of Veterinary Services at MDACC. Animals were anaesthetized using 2% isoflurane in oxygen (2 mL/min flow rate) and the fur was removed from the dorsum. Animals received a single injection of 0.1 mg/kg of buprenorphine prior to beginning the surgery. All surgical procedures were performed in aseptic conditions while maintaining body temperature at physiological levels using a heating pad. The skin was prepared by three alternating washes of chlorhexidine and 70% ethanol and ophthalmic ointment applied to the animal’s eyes. Before any incisions were made, the toe pinch reflex test was used to determine if the animal had attained a surgical level of anesthesia.

### 2.9. Dorsal Skin Window Chamber Implantation Surgery

Titanium dorsal skin window chambers were purchased from APJ Trading (APJ Trading Co, Inc, Ventura, CA, USA). The skin window chambers were modified by the UT MD Anderson Cancer Center Radiation Physics machine shop. The dorsal skin was drawn up into a longitudinal fold using a straight suture needle and sterile silk. The skinfold was trans-illuminated so that the symmetrical pattern of blood vessels on either side of the dorsal midline were matched for an even skinfold. The flat side of one of the window chamber pieces was held against the skin fold and the positions of the screw holes were marked. A hole was punched through both sides of the skin at each screw location using an 18 G needle. The front window chamber frame was then screwed into the rear frame and the top of the skinfold sutured along the edge of the frame through the skin to hold the window chamber in place. Antibiotic ointment was applied to all sites where screws and sutures passed through the skin. A 1.2 cm diameter circle in the top layer of the skin was cut with scissors along the circumference of the window chamber adjacent to the frame. The exposed dermis was washed with saline prior to an injection of 0.5 – 1 × 10^6^ tumor reporter cells between the exposed fascial plane and dermis. The exposed window area was then filled with saline until a meniscus formed; a glass cover slip was placed over the window, and secured with a retaining ring.

### 2.10. Abdominal Pancreas Window Chamber Implantation Surgery

The abdominal window chamber was produced by the UT MD Anderson Cancer Center Radiation Physics machine shop, custom modified using the specifications from Ritsma et al. [39] After window chamber preparation, described above, a sterile drape was placed over the animal with the surgery area remaining uncovered. Using a scalpel, a 15 mm incision through the skin on the dorsal left of the spine was made and the skin retracted. A similar length incision through the muscular layer was then made and the muscular tissue retracted, exposing the abdominal organs. The abdominal window chamber was held in position using a purse-string non-resorbable suture, which was concealed within the groove of the abdominal window chamber ring. A purse-string suture in the muscular layer was first made, followed by a purse-string suture through the skin. Concealing the suture within the groove of the ring prevented mice from biting or pulling the sutures. Once the abdominal window chamber was sutured in place, the pancreas was located and secured by gluing the organ to the interior inner side of the abdominal window chamber using high viscosity cyanoacrylates (Sigma-Aldrich, St. Louis, MO, USA). Once the pancreas was positioned within the abdominal window chamber, an orthotopic injection of 1 × 10^6^ reporter tumor cells was performed; note that no tumor cell injections were required for *KPC* or *KPC-Luc* animals. To further hold the organ in place, an optically translucent silicone seal (Kwik-Sil^TM^, World Precision Instruments, Sarasota, FL, USA) was placed around the pancreas. A 1.2 cm diameter glass cover slip was then placed on top of the window chamber and held in place by either glue or a retaining ring.

### 2.11. Post Window Chamber Implantation Surgery

After each window chamber implantation, the animal was placed on another heating pad and gently warmed during recovery; animals were injected subcutaneously with 1 mL of saline to maintain hydration and 0.1 mg/kg buprenorphine SR. Animals were monitored until fully recovered from the surgical procedure. Antibiotic ointment was applied along the tissue–implant interface. All animals were housed separately after window chamber implantation and the wire bar lid removed from the microisolator cages. A 2 mg Rimadyl tablet (Bio-Serv, Flemington, NJ, USA) was given orally in hydrogel and placed in the animal’s cage.

### 2.12. In Vivo Intravital Microscopy

To limit motion during intravital imaging, the UT MD Anderson Cancer Center Radiation Physics machine shop custom built window chamber imaging stage inserts made of aluminum and painted with black enamel that fit in the custom-crafted heated stage surface recess. The stage temperature was set to 37 °C to continuously warm animals during acquisition. Mice were kept under isoflurane anesthesia throughout the entire imaging session. To further minimize stray light during acquisitions, a small black box was placed over the animals with a notch cut out for the anesthetic nose cone. Intravital imaging generally occurred as follows: epifluorescence imaging of GFP or Dendra2 was performed (GFP cube) to locate and center the tumor. Next, confocal imaging was performed where indicated in which the manual shutter was placed in the open position to acquire confocal images. An i.p. injection of 300 mg/kg D-Luciferin was used for FLuc microscopy for single imaging sessions. Immediately following D-Luciferin administration, an epifluorescence image of GFP or Dendra2 occurred to ensure the tumor was still in focus. The confocal manual shutter was then pushed into the closed position and a bioluminescence image was taken 5 min post administration of D-Luciferin. Based on the 2X epifluorescence and bioluminescence images, an area of interest was magnified using either the 10X or 20X objective by epifluorescence or confocal imaging. When using the 10X and 20X objectives, image focus occurred using perfect focus to further prevent any drift from the focal plane. If an acquisition time of 10 min or less was used at 2X, a bioluminescence image at the higher magnification immediately followed. If an acquisition time of greater than 10 min occurred at 2X, a bioluminescence image occurred 5 min following a second i.p. injection of 300 mg/kg D-Luciferin.

### 2.13. Skin Window Chamber Intravital Microscopy

For imaging tumor signaling dynamics, an i.v. tail vein catheter was inserted and taped to the animal prior to being positioned on the microscope. The animal was placed on the microscope where the window chamber was locked in place using the custom stage insert. For imaging B16F10 κB-FLuc GFP reporter cells, an i.p. catheter was placed in the abdomen. Using the 2X objective, the tumor was located and centered using epifluorescence (GFP cube) and confocal (laser 488 nm) imaging. An i.p. injection of 300 mg/kg D-Luciferin occurred, followed by a steady infusion of D-Luciferin using a syringe pump at 8 µL/min to maintain a steady bioluminescence signal [25]. A basal bioluminescence image occurred at 5 min post i.p. injection of D-Luciferin at 2X (5 min exposure, open filter). Then, 10X epifluorescence (GFP cube) and confocal (4.8 speed, laser lines: 488 nm (Dendra2 excitation) and 561 nm (Texas Red excitation)) imaging occurred based on an area of interest chosen from the 2X bioluminescence image. A basal 10X bioluminescence image (10 min exposure, open filter) was taken. Following the basal bioluminescence image at 10X, live sequential C2 confocal imaging acquisition occurred and continued during the i.v. injection of dextran-Texas Red (10,000 MW, 4 mg/mL, ThermoFisher Scientific, Waltham, MA) to confirm a successful i.v. injection (*n* = 3 control animals, *n* = 4 TNFα treated animals). Animals were injected with dextran alone or a combination of dextran and TNFα. The total sequential C2 confocal acquisition time was 5 min. Following the successful i.v. injection of dextran with or without TNFα, sequential bioluminescence imaging (10X, 10 min exposure, open filter) occurred for 45 min. A final confocal image was taken following sequential bioluminescence imaging. The quantification of signaling dynamics occurred as follows: using the highest 10X bioluminescence image from the bioluminescence time series, multiple auto-detect ROIs were created around single cells or clusters of cells within the tumor. These ROIs were copied and pasted onto every bioluminescence image and counts were measured. All bioluminescence counts were normalized to the ROI area. Imaging *MPO^+/+^* (*n* = 14) and *MPO^−/−^* (*n* = 9) animals with B16F10 NF-κB-FLuc tumors occurred as follows: a Dendra2 fluorescence image (GFP cube) at 2X was acquired to locate the tumor, and then a bioluminescence image (20 min acquisition, open filter) occurred 5 min post i.p. injection of 300 mg/kg D-luciferin. The quantification of NF-κB transcriptional activation occurred as follows: an auto ROI was drawn around the tumor using the Dendra2 fluorescence image and copied to the corresponding bioluminescence image. Total counts were measured and normalized to ROI area. For vasculature leakage, live sequential confocal imaging acquisition (4.8 speed, laser lines: 488 nm (Dendra2 excitation) and 561 nm (Texas Red excitation)) occurred and continued during an i.v. administration of dextran-Texas Red (70,000 MW, 4 mg/mL, ThermoFisher Scientific, Waltham, MA) using the tail vein catheter. The total sequential C2 confocal acquisition time was 10 min. Quantification of dextran leakage occurred by drawing an ROI on a vessel and dextran fluorescence was measured as a function of time. The first image with measurable dextran fluorescence was set as time = 0, and the fluorescence intensity normalized to fluorescence intensity at time = 0 for all measured time points that followed to account for dextran delivery variability between animals (*n* = 4 animal per group). Normalized dextran leakage curves were fit using non-linear regression, least square fit. For in vivo imaging of myeloid cells, dextran-Texas Red (10,000 MW, 4 mg/mL, ThermoFisher Scientific, Waltham, MA) and αGr-1 Alexafluor647 (10 μg, BioLegend, San Diego, CA) were intravenously administered to label myeloid cells [9,40,41,42,43,44,45,46,47,48,49]. Live sequential confocal imaging acquisition (4.8 speed, laser lines: 488 nm (Dendra2 excitation), 561 nm (Texas Red excitation) and 640 nm (Alexfluor647 excitation)) occurred and continued during the intravenous injection of dextran and αGr-1 antibody. The total sequential C2 confocal acquisition time was 10 min. A region of interest was determined from the 2X confocal images to then image using the 10X objective. Five minutes post i.p. injection of 300 mg/kg D-Luciferin, a bioluminescence image was taken at 10X objective using a 20 min exposure (*n* = 4 *MPO^+/+^* animals). Following a 10X objective bioluminescence acquisition, a C2 confocal acquisition of the same region of interest at 10X occurred using the same acquisition parameters described above using a Z stack acquisition of 110 slices at 1 μm steps. Following intravital imaging, animals were given a bolus injection of 1 mL saline and recovered in their cage on a heating pad.

### 2.14. Photoswitching Dendra2 Fluorescent Protein

Single cell photoswitching in vivo was performed using the 405 nm laser on the confocal imaging system. Imaging acquisition occurred as follows: epifluorescence imaging (GFP cube) of Dendra2 located and centered the tumor at 2X. A bioluminescence image was acquired (2X, 10 min acquisition, open filter) 5 min post an i.p. injection of 300 mg/kg D-Luciferin. A bioluminescence image (10X, 20 min acquisition, open filter) followed, magnifying an area of interest chosen from the 2X bioluminescence image. Confocal imaging (4.8 speed, laser lines: 488 nm (green Dendra2 excitation) and 561 nm (red photoswitched Dendra2 excitation)) acquired a pre photoconversion image. An ROI was drawn around a cell to be photomarked. A live cell real-time sequential confocal sequence (4.8 speed, laser lines: 488nm and 561 nm) was acquired as follows: 5 confocal images prior to photoswitching, 20 confocal images acquired during stimulation with the 405 nm laser, and 20 confocal images post photoconversion. The 405 nm laser (3 stimulations, 20% power) photoswitched Dendra2 from green to red. Dendra2 photoswitching of larger tumor areas occurred as follows: epifluorescence imaging (GFP cube) of Dendra2 located and centered the tumor at 2X. A bioluminescence image was acquired (2X, 20 min acquisition, open filter) 5 min post an i.p. injection of 300 mg/kg D-Luciferin. Epifluorecence imaging (GFP and DsRed cubes) were performed at 20X; regions of interest were chosen based on the 2X bioluminescence image. The area of illumination for photoconversion was decreased to smaller than the field of view using the field stop slider. The target photoconversion area was exposed to the DAPI cube excitation light for 10 min to photoconvert Dendra2 at 20X. Epifluorescence imaging (GFP and DsRed cubes) at 20X and 2X occurred to confirm photoconversion. Imaging acquisition following single cell or tumor area Dendra2 photoconversion followed the general intravital imaging protocol described above at least 24 h later. For the quantification of Dendra2 photoconversion effects on bioluminescence reporter imaging, an auto ROI was drawn around the tumor using the Dendra2 epifluorescence image at 2X and copied to the corresponding bioluminescence image. Fluorescence and bioluminescence intensities were quantified on the day of, 24 h, and 48 h post photoconversion. Total quantified counts were normalized to the ROI area. Bioluminescence counts/area as well as bioluminescence counts/area normalized to fluorescence counts/area were calculated.

### 2.15. Spectral Unmixing

In vivo bioluminescence spectra were created using window chamber models with either B16F0 CBG or B16F10 FLuc tumors. Bioluminescence imaging occurred 5 min following a 150 mg/kg of body weight D-Luciferin using all emission filters (<492 nm, 540 ± 50 nm, and >594 nm filters, 5 min acquisition at all filters, 2X objective). Spectra for CBG and FLuc were generated using the analysis software, NIS-Elements AR Analysis (Nikon Instruments, Melville, NY, USA). Using all bioluminescence images with each emission filter, an auto threshold ROI was drawn around the tumor and a spectrum for each reporter was generated in each animal. These spectra were exported into excel where the mean spectrum was calculated (CBG, *n* = 5 images; FLuc, *n* = 4 images). The mean spectrum for each wavelength was saved as a csv file and uploaded into NIS-Elements software. Pure spectra for each reporter were found under a user-defined library when performing spectral unmixing. When spectral unmixing images, only the spectra for reporters that needed to be visually separated were selected. When quantifying the contrast to noise ratio (CNR), an auto threshold ROI was created on each spectrally unmixed BLI image. This ROI was copied onto each BLI image to quantify CNR. CNR was calculated using the following equation:

$$\mathrm{CNR}=\;\frac{\left({\text{mean bioluminescence}}_{\text{target}}-{\text{mean bioluminescence}}_{\text{non-target}}\right)}{{\text{standard deviation of bioluminescence}}_{\text{non-target}}}$$

### 2.16. Abdominal Window Chamber Intravital Microscopy

Bioluminescence imaging of the abdominal window chamber was described above in in vivo intravital microscopy. Vasculature imaging of abdominal window chamber animals occurred following an i.v. injection of dextran-FITC (200,000 MW, 4 mg/mL). The photoconversion of Dendra2 in abdominal window chambers occurred as described above in the photoswitching Dendra2 fluorescent protein methods section but using the Dendra2 photoswitching of larger tumor areas protocol.

### 2.17. Histological Analysis

Necropsy of animals occurred after 14 days, wherein the titanium window chamber was removed, skin excised and tissue frozen in OCT media. Prior to histological analysis, tumor samples were formalin fixed for 48 h and stored in 70% ethanol until histological staining. All tumor samples were sent to the Research Histology, Pathology and Imaging Core at UT MD Anderson Cancer Center where they were sectioned, stained and analyzed.

### 2.18. Statistical Analyses

Graphs were generated and statistical analyses were performed using GraphPad Prism (GraphPad Software, Inc, La Jolla, CA, USA). Data were expressed as mean ± s.e.m. Analysis of differences between two normally distributed paired test groups was performed using a Student’s *t*-test. Analysis of differences between unpaired groups was performed using an unpaired two-tail Mann–Whitney *t* test. *p* values were considered statistically significant if *p* < 0.05. For analysis of three or more groups, mixed-effects analysis were performed with Dunnett’s multiple comparisons test.

### 2.19. Data Availability

All relevant data are within the manuscript and the supplementary information files.

## 3. Results

### 3.1. Elimination of Light Sources by Custom Modification of an off-the-Shelf Imaging System

Bioluminescence imaging systems require a dedicated low-light system with a charge-couple device (CCD) camera; for microscopic bioluminescence imaging, a CCD camera is placed on the port of a microscopy system. Due to the low photon production of bioluminescence, eliminating stray light is imperative; the flux of stray light detectable by the camera should be <10X the rate of the minimum desired photonic flux to be measured by the camera. In this proof-of-principle demonstration project, our intravital microscopy system was assembled using the off-the-shelf Nikon TiE inverted microscope, a LSM C2 confocal system, and an Andor iKon-M 934 CCD camera. This microscopy system was assembled and housed in a separate dark room. Full characterization of the lower limit of detection and sensitivity of an integrated luminescence system and troubleshooting required an analysis of the detector itself (CCD camera) as well as the various sources of stray light. Ultimately, the elimination of key light sources resulted in a substantial net increase in signal-to-noise ratios.

The mean bias level from the CCD camera and stray light were determined with the camera shutter closed (Figure S1a). This represented the contribution of the dark current from the CCD camera to the mean bias and confirmed that the camera was behaving correctly with low noise. Under normal camera operations (≤−70 °C, binning 1 × 1), the camera yielded the expected linear response over time with the shutter closed. For dark current and stray light calculations, data were quantified as grey level/(sec _*_ pixel) and then converted into e^−^/(sec _*_ pixel) or photons/(sec _*_ pixel) by applying the vendor-supplied gain peak quantum yield for visible light. Data indicated that the dark current was within the expected tolerance for the camera. Utilizing 300 images (Figure S1b), the read noise was validated to be within specifications of the vendor. We were able to estimate the standard deviation and median average difference per pixel, indicating a very flat field with a minor manageable gradient across the sensor of <1 grey level or (1.2 e^−^) across the entire sensor surface. Other high-quality cameras from various vendors should display similar specifications.

**Figure 1:**
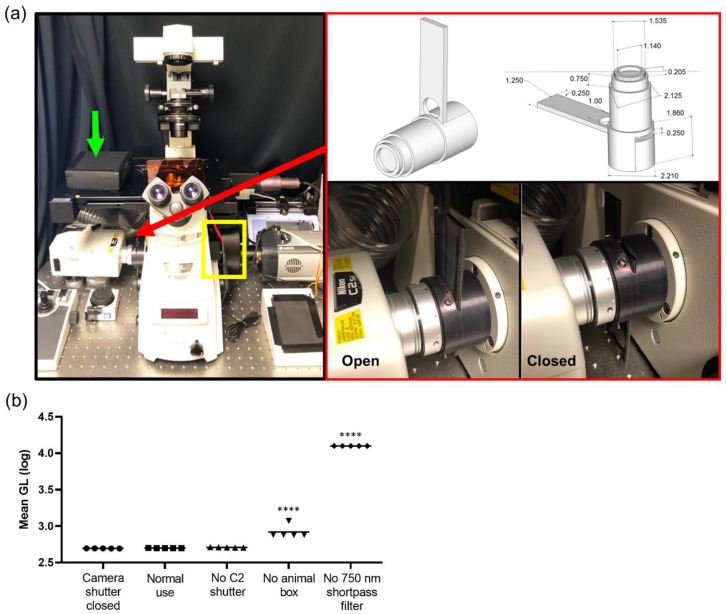
Intravital bioluminescence and fluorescence microscope configuration. (**a**) Photograph of microscope: charge-coupled device (CCD) camera on the right port, confocal system on the left port, yellow box indicates filter wheel, green arrow indicates black box placed over animal, and red arrow and red box show the manual shutter. (**b**) Quantification of light leakage throughout the system at every level of light elimination displayed with log scale (GL, grey level); **** *p* < 0.0001

Finding all internal light sources within the microscope was necessary as any and all light sources impact bioluminescence photonic acquisition. First, in the Nikon system, the internal reflection of NIR light from the real-time focal adjustment system was blocked using a 750 nm short pass filter placed within a filter wheel between the microscope and CCD camera (Figure 1a, yellow box). Next, when combining bioluminescence imaging with confocal imaging, the interlock system of the confocal system has an internal light source that needs to be blocked from the CCD camera. A manual shutter was created that blocks any internal light leak from the confocal system (Figure 1a, red arrow and box). Furthermore, a moveable black box was placed over the animal on the microscope stage (Figure 1a, green arrow) as well as blackout curtains surrounding the entire microscopic system were installed, blocking any light from the computer system controlling the microscope or outside stray light sources. Quantifying light leakage throughout the system demonstrated that every level of elimination of stray light was necessary to lower the background photon count (Figure 1b). Removing the 750 nm short pass filter that blocks the internal NIR light from the indwelling focusing system resulted in the largest increase in the bias. Suppression of the NIR stray light with the 750 nm short pass filter suppressed the transmission of stray visible light from the sample to the detector. The vendor-supplied specifications indicated a <10% loss of visible light when the filter was in place, and indeed, when measured with a luminescence light standard, we quantified an approximately 6.5% loss of signal, but with a concomitant 2700% decrease in the noise, netting an approximate 25-fold increase in sensitivity (Figure S1c,d). Since this system was utilized to quantify bioluminescence signals throughout tumor development, day to day reproducibility of measurements was validated. Using a light standard, the light measured on day 0 was plotted against the light measured on day 1 (Figure S1e). The data points were fitted with a line displaying a slope of 1, indicating excellent day-to-day test–retest agreement; Lin’s concordance correlation co-efficient was 0.9968. A stress test was further conducted to quantify any residual stray light. The integrated system yielded a uniform background when stress that was tested was, on average, 1% ± 2.1% different from the detector noise. Median images were acquired using 20 min acquisitions, comparing the camera noise (pixel-wise dark current when the camera shutter was closed) with normal use (shutter open) (Figure S1f). The percentage difference per pixel was calculated and displayed as an image. There was clearly <10% difference across the field.

### 3.2. In Vivo Dorsal Skin Window Chamber Intravital Bioluminescence Imaging

**Figure 2:**
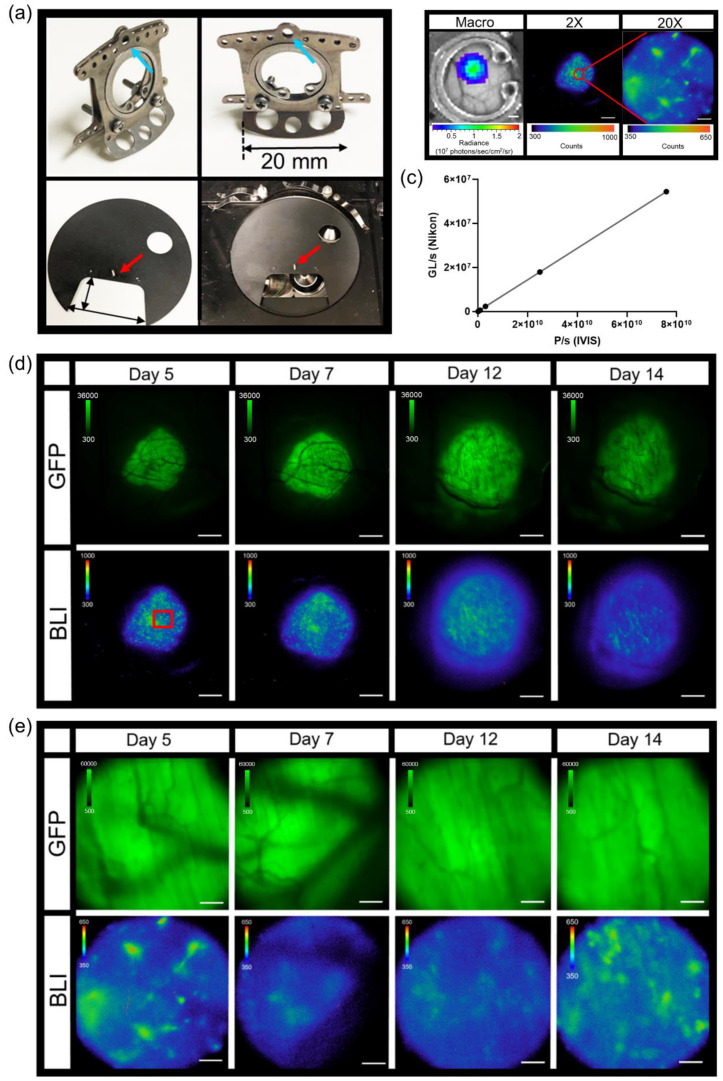
Intravital dorsal skin window chamber bioluminescence and fluorescence imaging. (**a**) Modified skin window chamber implant where front plate flange was shorted to 20 mm (top photos) and custom imaging stage insert (bottom photos; 11 cm diameter insert with a 5.5 cm × 3 cm cutout for the animal to lay flat). A pin protruding perpendicular to the custom imaging stage insert (red arrow) fits into the window chamber implant (blue arrow) to further lock the animal in place during imaging. (**b**) Representative bioluminescence images of B16F10 κB5-FLuc GFP tumors in skin window chamber-bearing animals using the macro-imaging system (macro, scale bar represents 1000 µm) and the microscopy system at 2X (5 min acquisition, open filter, scale bar represents 1000 µm) and 20X (10 min acquisition, open filter, scale bar represents 100 µm) where the red circle on the 2X image indicates a magnified area. (**c**) Linearity analysis between the macro-imaging system (IVIS) and micro-imaging system (Nikon); the correlation was measured at 1 (*p* < 0.0001) using *n* = 5 images where data were expressed as mean ± s.e.m. (error bars fall within the data point). Representative in vivo microscopic imaging over time of B16F10 κB5-FLuc GFP reporter tumor growth wherein GFP monitors B16F10 tumor growth and BLI monitors FLuc bioluminescence indicative of transcriptional activation of NF-κB at (**d**) 2X magnification (GFP cube, 500 ms; BLI, 5 min acquisition, open filter, scale bar represents 1000 μm, red box is corresponding area imaged at 20X) and (**e**) 20X magnification (GFP cube, 20 ms; BLI, 10 min acquisition, open filter, scale bar represents 100 μm).

A modification of the standard dorsal skin window chamber as well as a custom imaging stage insert were fabricated in order to limit the movement of the window chamber and assure that the cover glass remained level during microscopy (Figure 2a). The bottom flange of the front piece of the custom window chamber was shorten to 20 mm in length (Figure 2a, top pictures). The shortened window chamber did not affect the stability of the implant on the animals. An 11 cm diameter custom imaging stage insert was fabricated with a 5.5 × 3 cm recess cutout that allowed skin window chambers to be inserted below the stage neutral plane, such that the cover glass could lay flat at the focal plane (Figure 2a, bottom pictures). A pin placed adjacent to the recess (cutout) in the custom imaging stage insert (Figure 2a, bottom pictures, red arrow) fit the middle top hole of the window chamber implant (Figure 2a, top pictures, blue arrows), which limited window chamber motion during microscopy. A 2 cm diameter circle window was cut in the custom stage insert that allowed the user to see which objective was in place during window chamber mouse imaging.

Combination fluorescence and bioluminescence intravital microscopy was demonstrated using skin window chamber-bearing animals injected with B16F10 melanoma cells stably expressing a concatenated κB5 promoter-driven Firefly luciferase reporter (κB5-FLuc) [30,36] that monitors NF-κB activation and a constitutively active IRES promoter-driven GFP fluorescent reporter. Tumor growth was monitored by GFP imaging and NF-κB transcriptional activation was imaged by bioluminescence. Macro-imaging of bioluminescence a day prior to bioluminescence microscopy occurred first (Figure 2b, Macro). Note that the bioluminescence macro-image demonstrated the highest NF-κB transcriptional activation at the center of the tumor mass, which decreased radially [32]. When the same animals were imaged by bioluminescence microscopy, surprisingly, the activation of NF-κB was heterogeneous within the B16F10 tumor; macro-BLI imaging did not capture the heterogeneity in reporter signaling that the microscopic platform readily imaged (Figure 2b, 2X and 20X). The field of view of the 2X objective demonstrated high linearity between the widely utilized macro-imaging systems (IVIS Spectrum) and our custom micro-imaging system (Figure 2c); the correlation was measured at 1 (*p* < 0.0001). Sensitivity comparisons between the macro- and micro-imaging systems indicated that, when object radiance was >10^6^ photons/sec/cm^2^/sr on the macro-imaging system, the visualization of micro-structures utilizing the microscope with < 20 min acquisition times was achieved. Thus, while highly linearly correlated, the sensitivity differences between the two systems could be attributed to changes in image pixel size and the effective f stop, even when the largest NA 2X objective was used.

Although the use of the C2 manual shutter decreased the photon count by only 10.6 ± 2.2 grey levels when quantifying light leakage of the microscopy system (Figure 1b,c), this significantly impacted bioluminescence imaging of NF-κB activation in skin window chamber B16F10 tumor-bearing animals (Figure S1g). When the C2 shutter was open, light leakage from the confocal system contaminated the bioluminescence image compared to when the C2 shutter was closed (Figure S1g). As an application example, skin window chamber B16F10 κB5-FLuc tumor-bearing animals were imaged over time at both 2X and 20X magnification (Figure 2d,e). As expected, GFP fluorescence increased over time, indicative of an increase in tumor size. As tumors progress, vasculature also develops, which was observed in the GFP images as dark reticulated shadows. NF-κB-driven bioluminescence also increased as the tumor increased, wherein the extensive spatial and temporal heterogeneity of bioluminescence was observed throughout tumor progression. Using the 20X objective, single tumor cells or small clusters of tumor cells in vivo were readily resolved by bioluminescence, wherein NF-κB transcriptional activation could be quantified more precisely and were shown to change spatially and temporally during tumor development (Figure 2e and Figure S2a).

### 3.3. Imaging and Quantification of Tumor Signaling Dynamics

**Figure 3:**
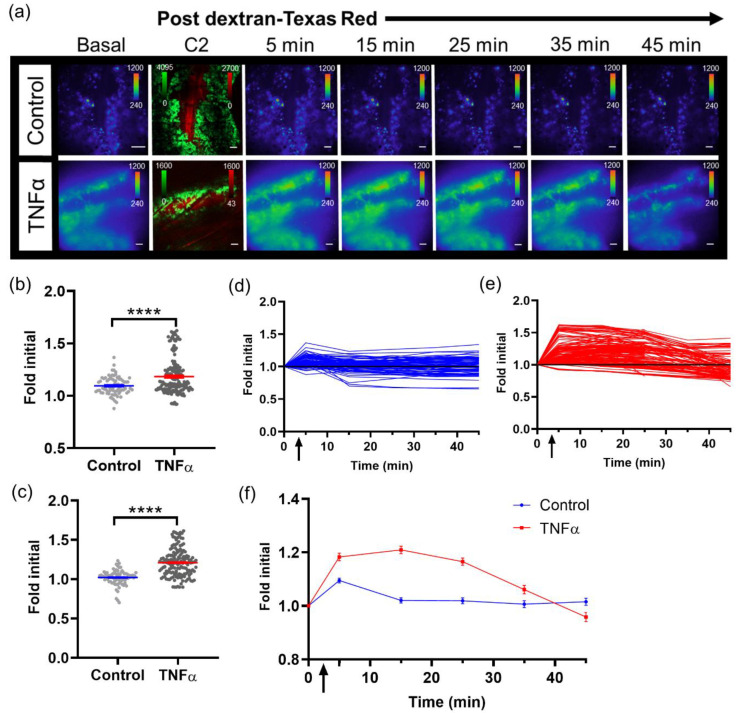
Imaging single tumor cell signaling dynamics in vivo. (**a**) Representative in vivo microscopic images over time of NF- κB transcriptional activation by TNFα in B16F10 κB5-FLuc GFP reporter tumor cells in skin window chamber-bearing animals; 10X magnification; BLI, 10 min acquisition, open filter; Confocal imaging (C2), 4.8 speed, laser lines: 488 nm (GFP, tumor) and 561 nm (dextran-Texas Red, vasculature); scale bar represents 100 μm. BLI images showing NF- κB activation were obtained prior to and following dextran-Texas Red i.v. injection without (control, *n* = 3 animals) or with TNFα (*n* = 4 animals) at multiple time points (Basal, 5, 15, 25, 35, 45 min), while confocal image (C2) confirms presence of tumor cells (GFP, green) and successful i.v. administration of dextran-Texas Red (red). Quantification of changes in bioluminescence, indicative of changes in NF-κB transcriptional activation, following dextran alone or with TNFα administration at (**b**) 5 min and (**c**) 15 min (**** *p* < 0.0001). Quantification of bioluminescence changes over time in individual tumor cells in animals administered (**d**) dextran, or (**e**) dextran with TNFα. (**f**) Quantification of mean NF-κB transcriptional activation within tumor cells over time in vivo (data are represented as mean ± SEM); black arrow indicates when dextran alone or dextran with TNFα were administered.

With the capability to image tumor cell bioluminescence in vivo, the NF-κB transcriptional activation of B16F10 κB5-FLuc tumors were imaged over time with and without TNFα activation. A basal bioluminescence image was first imaged at 10X (Figure 3a, Basal). As a delivery control, sequential confocal imaging confirmed the successful intravenous injection of dextran alone or with TNFα (Figure 3a and Figure S2b). Next, sequential bioluminescence imaging monitored the induced NF-κB transcriptional activation following dextran without or with concurrent TNFα administration (Figure 3a, 5–45 min images). NF-κB transcriptional activation was quantified by measuring the bioluminescence signal in individual tumor cells within individual animals at all imaged time points. Bioluminescence signals were normalized to the basal bioluminescence signals to account for variability in initial NF-κB transcriptional levels in individual tumor cells and between animals. At both 5 min and 15 min post concurrent administration of dextran and TNFα, TNFα activation significantly increased the NF-κB transcriptional bioluminescence signal, respectively (Figure 3b,c). Single cells and/or clusters of cells were quantified within control and TNFα-activated skin window chamber B16F10 κB5-FLuc tumor-bearing animals over time (Figure S2c). Fold initial signal (normalized to basal signal) demonstrated variability between individual tumor cells as well as between control and TNFα-treated animals (Figure 3d,e). However, overall, the mean of quantified NF-κB transcriptional activation within tumor cell populations over time demonstrated an increase in bioluminescence in TNFα-treated animals compared to control animals that lasted for approximately 40 min (Figure 3f).

**Figure 4:**
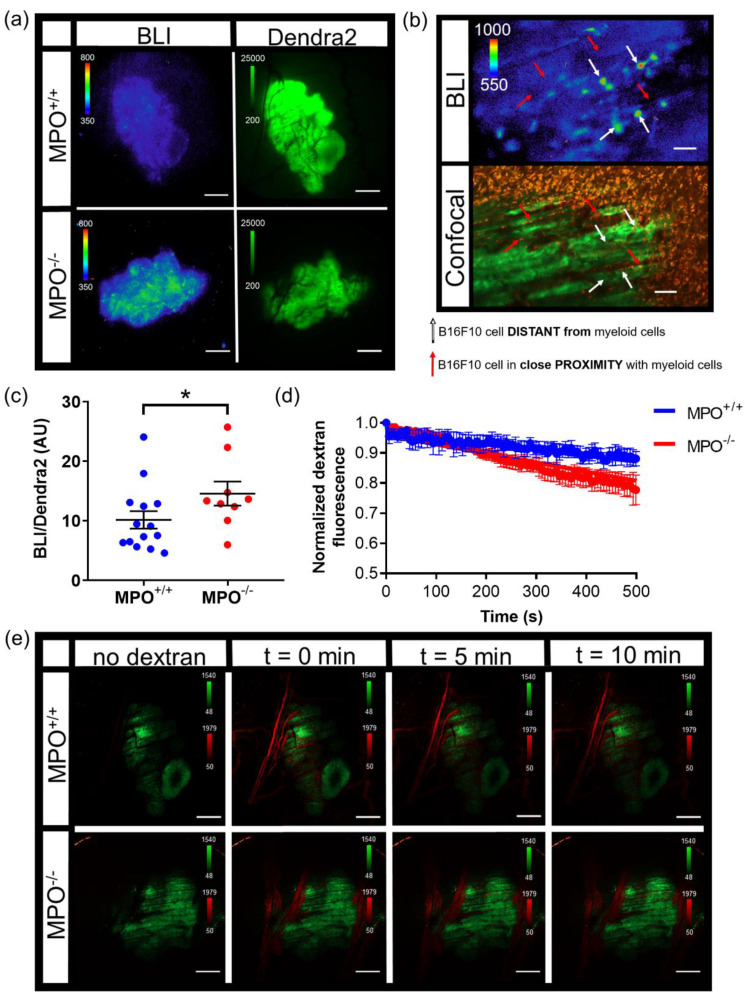
Intravital microscopy of wild type (*MPO^+/+^*) and *MPO*-null (*MPO^−/−^*) window chamber-bearing animals with B16F10 NF-κB-FLuc Dendra2 melanoma tumors. (**a**) Representative intravital image of NF-κB-FLuc bioluminescence (BLI, 20 min acquisition, open filter) and Dendra2 epifluorescence (GFP cube, 1 s exposure) at 2X in *MPO^+/+^* and *MPO^−/−^* animals at day 7 post window chamber implantation and tumor inoculation; scale bar represents 1000 μm. (**b**) Representative high resolution intravital microscopic image of bioluminescence (BLI) and corresponding confocal image of B16F10 NF-κB-FLuc tumor cells (Dendra2; green), myeloid cells (αGr-1+; red, and dextran uptake; orange); red arrows indicate B16F10 cells in contact with myeloid cells, white arrows indicate B16F10 cells not in contact with myeloid cells, 10X objective; scale bar, 100 μm (alternate image of previously reported publication [50]). (**c**) Quantification of NF-κB-FLuc bioluminescence normalized to tumor Dendra2 fluorescence between day 5 and day 7 post tumor inoculation (data are represented as mean ± SEM, statistical significance calculated by unpaired two-tail Mann–Whitney *t* test, * *p* < 0.05, *n* = 14 *MPO^+/+^* animals, *n* = 9 *MPO^−/−^* animals). (**d**) Quantitative decrease in Texas Red fluorescence corresponding to leakage of dextran from the vasculature of B16F10 tumors over time (data are represented as mean ± SEM, *n* = 4 animals per group). Dextran leaks out of *MPO^−/−^* B16F10 tumor vasculature significantly faster than from *MPO^+/+^* B16F10 tumor vasculature (least squares fit, slope significantly different, *p* < 0.0001). (**e**) Representative confocal images in *MPO^+/+^* and *MPO^−/−^* animals of tumor vasculature post i.v. injection of dextran-Texas Red (70,000 MW, 4 mg/mL) at different time points (green, B16F10 Dendra2 fluorescence; red, dextran-Texas Red); 2X objective, scale bar indicates 1000 μm.

NF-κB transcriptional activation within melanoma tumors were compared in wild type (*MPO^+/+^*) and syngeneic *MPO*-deficient (*MPO^−/−^*) animals with skin window chambers. B16F10 melanoma cells stably expressed a NF-κB promoter-driven FLuc reporter (B16F10 NF-κB-FLuc) where the FLuc bioluminescence magnitude corresponded to the level of NF-κB transcriptional activation within the melanoma cells (Figure 4a, BLI). Similar to the B16F10 κB5-FLuc tumors, heterogeneous basal NF-κB transcriptional activation within individual tumors and tumor cells was observed at both 2X and 20X (Figure 4a,b, BLI). B16F10 tumor cells also stably expressed a Dendra2 fluorophore to fluorescently locate and monitor tumor growth (Figure 4a, Dendra2; Figure S2d). The bioluminescence signal from B16F10 NF-κB-FLuc tumors were quantified at 2X between day 5 to day 7 post tumor inoculation. The inclusion of a constitutively active reporter, such as Dendra2Dendra2, provided a method to normalize signaling dynamics to tumor mass. Normalized NF-κB activation per unit tumor mass (Figure 4c) demonstrated an increase in NF-κB transcriptional activation within B16F10 tumors grown in *MPO^−/−^* animals compared to *MPO^+/+^* animals, which had been previously macroscopically demonstrated [50]. To explore whether the spatial heterogeneity of NF-κB transcriptional activation was a consequence of myeloid cell distribution within the microenvironment, myeloid cells were labeled in vivo by endocytosis using an intravenous injection of fluorophore-labeled dextran (orange) as well as by a surface marker using fluorophore-labeled αGr-1 antibody (red) [9,40,41,42]. Remarkably, tumor cells (green) in close proximity to myeloid cells (red) in vivo demonstrated decreased NF-κB-FLuc bioluminescence (Figure 4b, red arrows) compared to tumor cells that were distant from myeloid cells (Figure 4b, white arrows). The histology of window chamber skin flaps at endpoint confirmed the presence of myeloid cells (Figure S2e). In addition, vasculature permeability was assessed following an intravenous injection of fluorophore-labeled dextran in real-time (Figure 4d,e). The decrease in fluorescence corresponded to the leakage of dextran from the vasculature within B16F10 tumors. Interestingly, the leakage of dextran was significantly faster from B16F10 tumors in *MPO^−/−^* animals compared to *MPO^+/+^* animals suggesting that the vasculature was more permeable in melanoma tumors grown in *MPO*-deficient environments (Figure 4d,e). Importantly, these in vivo window chamber studies demonstrated the technical advance and ability to image and quantify NF-κB signaling dynamics within tumors in vivo over time during tumor progression, combining both fluorescence and bioluminescence microscopic imaging within the identical field of view.

### 3.4. Photoswitchable IVM Imaging

**Figure 5:**
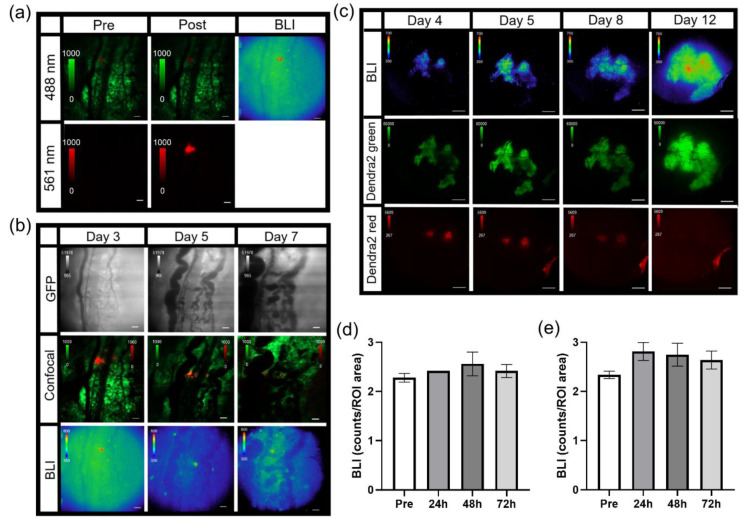
Photoswitching B16F10 NF-κB-FLuc Dendra2 tumor cells and tumor areas. (**a**) Representative intravital confocal images (4.8 speed, laser lines: 488 nm (green unphotoconverted Dendra2) and 561 nm (red photoconverted Dendra2)) pre and post single cell photoconversion using the 405nm laser line and corresponding bioluminescence image (BLI, 20 min acquisition, open filter) at 10X; scale bar represents 100 μm. (**b**) Representative intravital microscopic image of B16F10 NF-κB-FLuc Dendra2 tumors over time following 405 nm confocal laser photoconversion of Dendra2 epifluorescence (GFP cube, 200 ms exposure), merged Dendra2 confocal image (green unphotoswitched and red photoswitched Dendra2) and NF-κB-FLuc bioluminescence image at 10X. (**c**) Representative intravital image of B16F10 NF-κB-FLuc Dendra2 tumors over time following photomarking larger tumor areas at 2X of NF-κB-FLuc bioluminescence (BLI), green unphotomarked Dendra2 (Dendra2 green, GFP cube, 2 s exposure), and red photomarked Dendra2 (Dendra2 red, DsRed cube, 5 s exposure). Quantification of NF-κB-FLuc bioluminescence prior to (pre) and at indicated time points post Dendra2 photoconversion of (**d**) whole tumor, and (**e**) photoconverted areas of the tumor (data are represented as mean ± SEM, *n* = 6 animals).

The Dendra2 fluorophore was developed as a photoswitchable green-to-red fluorescent protein [51]. For this example, window chamber B16F10 NF-κB-FLuc Dendra2 tumor-bearing animals were imaged by bioluminescence and confocal microscopy on day 3 post tumor inoculation (Figure 5a). Under control conditions, non-photoconverted Dendra2 fluoresced when excited using the 488 nm laser, and showed no detectable fluorescence using the 561 nm laser (Figure 5a, Pre). Using the bioluminescence image, a cell of interest with high NF-κB transcriptional activation was chosen to be photomarked (Figure 5a, BLI). Following photoconversion using the 405 nm laser, Dendra2 was photoswitched to red fluorescence and the cell was now detectable using the 561 nm laser (Figure 5a, Post). Photoconverted cells were detectable for several days; however, by day 7 (4 days after photoconversion), the red Dendra2 signal had drastically decreased (Figure 5b, Confocal). Importantly, the bioluminescence of photomarked cells could be followed over time and demonstrated changes in NF-κB transcriptional activation at different imaging time points (Figure 5b and Figure S3a). The photoconverted cells were detectable at 2X immediately following photoswitching as well as 2 days later (day 5); however, again, by day 7, the red Dendra2 fluorescence was too weak to be detected at 2X (Figure S3b). Larger areas of a tumor stably expressing Dendra2 could also be photoconverted using the DAPI cube of the epifluorescence system. Herein, green Dendra2 was detected on the epifluorescence system using a GFP filter cube and red photoswitched Dendra2 was detected using a DsRed filter cube (Figure 5c). By photoswitching larger areas, the red Dendra2 fluorescence was detectable for longer periods of time, e.g., 7 days post photoconversion. Importantly, the inclusion of a bioluminescent reporter did not affect the photoswitching properties of Dendra2 nor did photoconversion of Dendra2 within tumors affect the bioluminescent reporter. For example, following the photomarking of larger areas of B16F10 NF-κB-FLuc Dendra2 tumors, quantification of bioluminescence demonstrated no significant change in total tumor NF-κB transcriptional activation over time (Figure 5d and Figure S3c) or bioluminescence in photoswitched areas of the tumor (Figure 5e and Figure S3d). When bioluminescence was normalized to Dendra2 fluorescence, by total tumor (Figure S3e) or only in photoswitched areas of the tumor (Figure S3f–h), no significant change was demonstrated when bioluminescence in the photoswitched areas of the tumor was normalized to the photoconverted red Dendra2 (Figure S3h). However, a decrease in the NF-κB transcriptional activation bioluminescence was observed when normalized to total Dendra2 (unphotoconverted green + photoconverted red Dendra2) or unphotoswitched green Dendra2 fluorescence (Figure S3e–g). These data demonstrated the difficulties in using Dendra2 fluorescence quantification following photoconversion for bioluminescence signal normalization because the photoconversion of Dendra2 is non-reversible [51]. Thus, differences in fluorescence intensities occur between unphotoconverted green and photoconverted red Dendra2; loss in green Dendra2 fluorescence was not conserved by a gain in photoconverted red Dendra2 fluorescence [51]. Furthermore, photoswitched red Dendra2 fluorescence decreased over time. Nonetheless, the advantage of using a photoswitchable fluorophore allowed cells or areas of interest to be marked and followed throughout significant time intervals over tumor progression. Here, the change in NF-κB transcriptional activation of either single cells or specific areas of tumors were monitored following photoconversion of Dendra2 from green to red.

### 3.5. Generation of Spectra In Vivo for IVM

**Figure 6:**
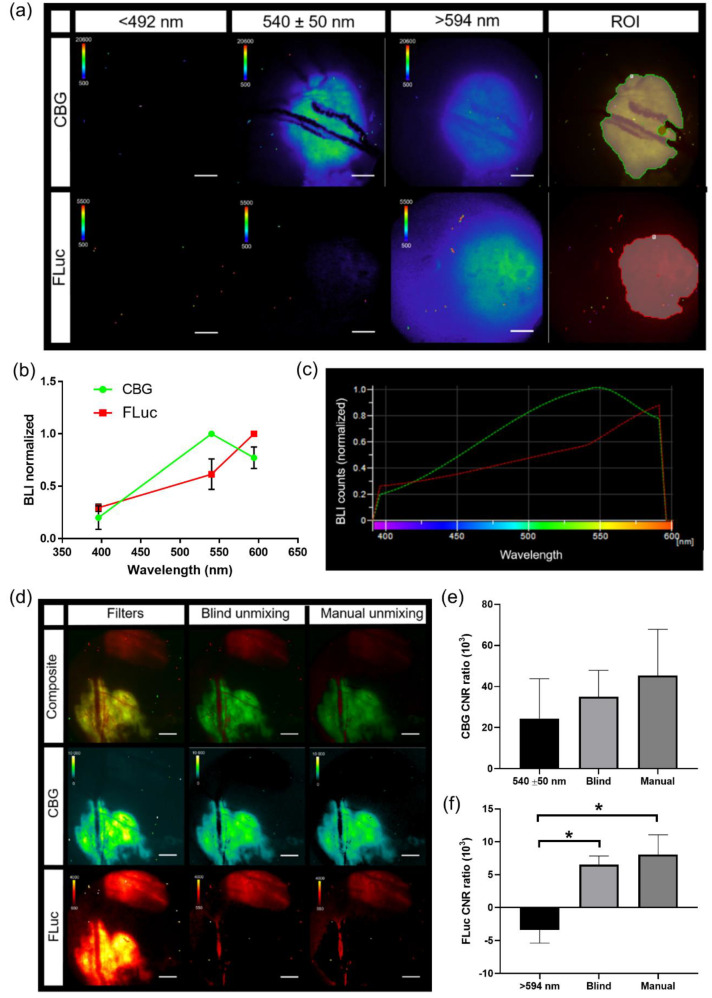
Generation of pure in vivo bioluminescence spectra for spectral unmixing. (**a**) Representative intravital micro-bioluminescence images of individual animals with B16F10 tumors stably expressing either click beetle green (CBG) or Firefly luciferase (FLuc) (BLI, 5 min acquisition, <492 nm, 540 ± 50 nm, and >594 nm filters, 2X objective; scale bar represents 1000 μm) and auto ROI drawn for collection of pure spectra (ROI). (**b**) Normalized in vivo emission spectra for different bioluminescent reporters, and (**c**) imported user-generated spectral library to unmix CBG and FLuc (*n* = 5 animals imaged with CBG, *n* = 4 animals imaged with FLuc). (**d**) In vivo bioluminescence unmixing of B16F10 tumors; one B16F10 tumor stably expressed ubiquitin-CBG and the other B16F10 tumor stably expressed CMV-FLuc. Representative in vivo bioluminescence image of B16F10 CBG spectrally unmixed from B16F10 FLuc tumors in the same animal within the same window chamber using imaging filters alone (Filters), commercial blind unmixing built into the NIS-Elements software (blind unmixing) and manual unmixing using user-generated spectral library (manual unmixing) following a single i.p. injection of D-Luciferin (BLI, 5 min acquisition, 540 ± 50 nm and >594 nm filters, 2X objective; scale bar represents 1000 μm). B16F10 CBG was spectrally separated from B16F10 FLuc wherein the composite image shows pseudo-colored B16F10 CBG (green) and B16F10 FLuc (red) tumors within the same window chamber (*n* = 4 images (2 animals, 2 imaging time points)). Contrast to noise ratio (CNR) of unmixed signal for (**e**) CBG and (**f**) FLuc using filters alone, blind unmixing and manual user-generated spectra (*n* = 4 images; * *p* < 0.05).

Spectral bioluminescence unmixing techniques have been described previously for in vitro and for macro-imaging applications [31,32,33,34,35]. Similarly, in order to apply spectral unmixing to studies in vivo, pure spectra of each BLI reporter must first be generated for the microscopy system. Skin window chamber tumor-bearing animals were injected with B16F10 melanoma cells: either a B16F10 cell line that stably expressed an ubiquitin promoter-driven click beetle green (CBG, λ_em_ = ~540 nm in vivo) reporter or a B16F10 tumor cell line that stably expressed a CMV promoter-driven firefly luciferase (FLuc, λ_em_ = ~600 nm in vivo) reporter. Skin window chamber tumor-bearing animals were imaged using three different filters: 492 short pass (<492 nm), 540/50 band pass (540 ± 50 nm), and 594 long pass (>594 nm) (Figure 6a). A spectrum was measured from the emission filter images for CBG and FLuc from individual imaging sessions and a mean spectrum for each reporter was generated (Figure 6b) and imported into the microscope software (Figure 6c). To validate that the user-generated pure spectral library correction algorithm was capable of unmixing CBG from FLuc simultaneously, window chamber-bearing animals were inoculated with B16F10 CBG (green) and B16F10 FLuc (red) cells in different areas of the window. Following a single D-luciferin i.p. injection, bioluminescence imaging with the 3 emission filters was performed. CBG expressing tumors could be spectrally umixed from FLuc expression tumors when processed with our pure spectral library correction algorithm (Figure 6d, Manual). It should be noted that CBG photons emitted under the vasculature were red-shifted due to absorption and/or scattering with hemoglobin and captured in the FLuc channel. However, the vasculature can be easily imaged by brightfield or fluorescence and can be masked from the BLI image when quantifying photon flux. Although it was possible to unmix the CBG tumor from the FLuc-expressing tumor with the emission filters alone (Figure 6d, Filters) and the commercially built-in blind unmixing algorithm (Figure 6d, Blind umixing) available in the software, the contrast-to-noise ratio (CNR) was further improved when using our user-generated pure spectral library correction algorithm, especially in the FLuc channel (Figure 6e,f). The CNR for CBG was 24.4 ± 19.3, 35.0 ± 12.8 and 45.3 ± 22.5 for the 540 nm filter alone, built-in blind unmixing and using our user-generated pure spectral library correction algorithm, respectively, while the CNR for FLuc was −3.40 ± 1.98, 6.52 ± 1.33 and 8.04 ± 3.01 for the >594 nm filter alone, blind unmixing, and using our spectral library, respectively. The highest CNRs in both the CBG and FLuc channel occurred when images were unmixed with our user-generated pure spectral library correction algorithm, which demonstrated an enhanced signal separation afforded by the user-generated pure spectra corrections.

### 3.6. IVM of Abdominal Window Chamber Tumor-Bearing Animals

**Figure 7:**
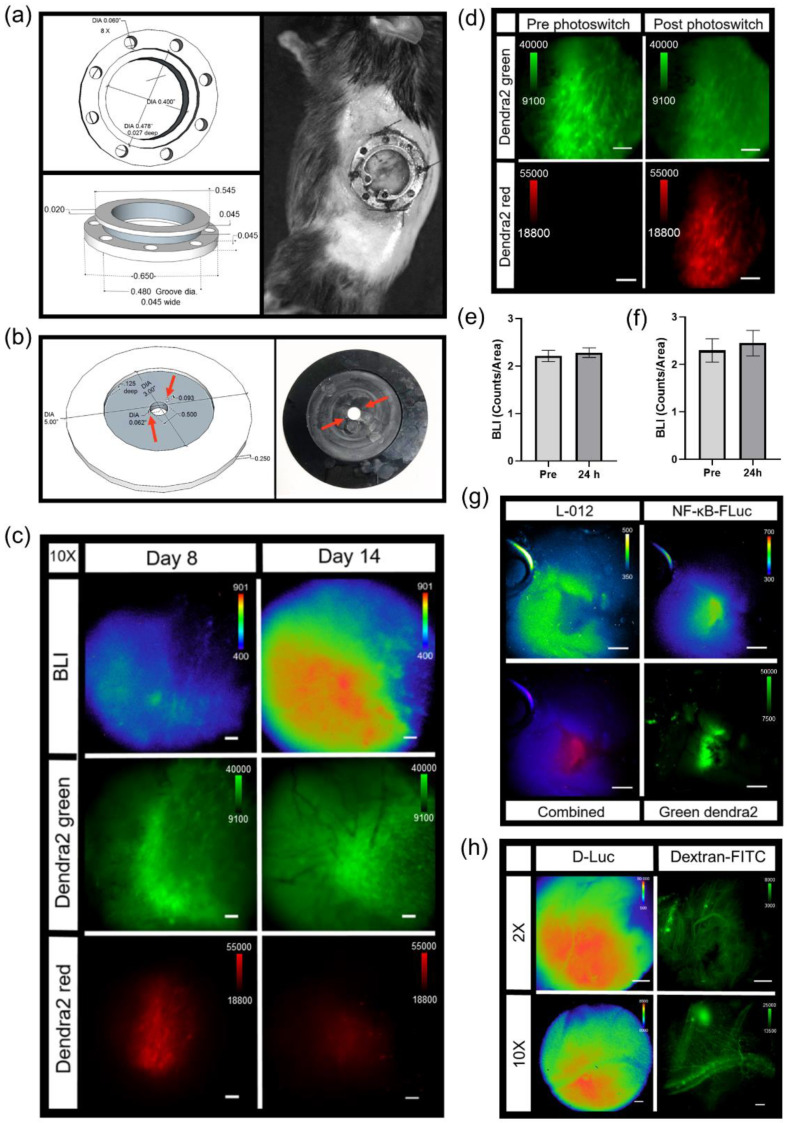
Intravital bioluminescence and fluorescence microscopy of pancreas window chamber. Diagram with measurements and representative photograph of (**a**) pancreas window chamber and (**b**) imaging stage insert. (**c**) Intravital microscopy images of the pancreas using wild type C57BL/6 abdominal window chamber-bearing animals with Pan02 NF-κB-FLuc Dendra2 pancreatic tumors. Representative intravital image of NF-κB-FLuc bioluminescence and Dendra2 fluorescence of Pan02 pancreatic tumor progression over time at 10X objective (BLI, 10 min acquisition, open filter; Dendra2 green, GFP cube, 200 ms exposure; Dendra2 red, DsRed cube, 500 ms; scale bar represents 100 μm). (**d**) Representative fluorescence image of Pan02 tumor pre- and post-photoswitching (DAPI cube, 10 min exposure) at 10X objective (Dendra2 green, GFP cube, 200 ms exposure; Dendra2 red, DsRed cube, 500 ms; scale bar represents 100 μm). Quantification of NF-κB-FLuc bioluminescence counts of (**e**) total tumor and (**f**) photoconverted area of the tumor pre- and 24 h post-Dendra2 photoconversion (data are represented as mean ± SEM, *n* = 3 animals). (**g**) Representative intravital images of resolved bioluminescence of L-012 innate immune cell activity and Pan02 NF-κB-FLuc reporters (2X objective, sequential imaging of L-012 (20 min acquisition, open filter) and NF-κB-FLuc (10 min acquisition, open filter)), overlaid image of bioluminescent reporters (combined; pseudocolored red), and tumor Dendra2 (Dendra2 green, GFP cube, 2 s exposure); scale bar represents 1000 µm. (**h**) Representative intravital images of abdominal window chamber implanted at week 14 visualizing the pancreas of *KPC-Luc* animals at 2X (FLuc, 2 min acquisition, open filter; scale bar represents 1000 µm) and 10X (FLuc, 2 min acquisition, open filter; scale bar represents 100 µm); *n* = 2 animals. Pancreas tumor growth monitored by FLuc bioluminescence following D-luciferin injection. Vasculature imaged post i.v. injection of dextran-FITC (200,000 MW, 4 mg/mL, epifluorescence: GFP cube; 2X, 5 s exposure; 10X, 2 s exposure).

An abdominal window chamber was custom fabricated, modified from the specifications of Ritsma et al. [39] (Figure 7a). The eight holes in the implant were used for sutures to secure the window chamber on the animal, but also used to lock the window chamber in place during microscopy. This was accomplished by using a custom imaging stage insert, which contained two notches 180° apart (Figure 7b, red arrows). These notches fit into corresponding holes in the abdominal window chamber to eliminate motion during imaging. During window chamber implantation, the orthotopic injection of pancreatic tumor cells was performed. The pancreatic cancer cell, Pan02, stably expressed both the NF-κB transcriptional activation bioluminescent reporter and a constitutively active CMV promoter-driven Dendra2 fluorophore (Pan02 NF-κB-FLuc Dendra2). Similar to the skin window chamber, the basal heterogeneity of tumor NF-κB trp-21anscriptional activation was only observed when using bioluminescence microscopy (Figure 7c and Figure S4a); macro imaging of Pan02 NF-κB-FLuc Dendra2 tumors in abdominal windows demonstrated the radial distribution in the signal, also seen in skin window chambers (Figure S4a, macro). NF-κB activation by bioluminescence and tumor growth by Dendra2 fluorescence were monitored over time at both 2X (Figure S4a) and 10X (Figure 7c). The photoconversion of Dendra2 was achieved and detectable (Figure 7c,d and Figure S4b). Photomarked Dendra2 was detectable at 7 days post photoconversion (Figure 7c,d). Quantification of the bioluminescence NF-κB signal from tumor cells (Figure 7e) and of the photoswitched area (Figure 7f) was similar pre- and post-Dendra2 photoswitching. Thus, again, the NF-κB transcriptional activation reporter did not affect the photoconversion of Dendra2 nor did photoswitching Dendra2 affect the bioluminescent reporter. Additionally, the exogenous bioluminescent reporter L-012, known to image innate immune infiltration based upon myeloperoxidase activity, reactive oxygen species and reactive nitrogen species production [52], was detected by bioluminescence microscopy (Figure 7g). Using sequential imaging sessions, innate immune infiltrate (L-012) was easily resolved in the same animal from tumor NF-κB transcriptional activation (FLuc) due to the different substrates used. L-012 bioluminescence was further resolved from FLuc bioluminescence by using the red filter to detect the FLuc bioluminescence (Figure S4c). As expected, NF- κB-FLuc bioluminescence overlapped with Dendra2 fluorescence, since both reporters were expressed only in tumor cells. L-012 imaging demonstrated that different animals had different innate immune cell infiltration accumulation at the same time point post tumor inoculation; one animal demonstrated innate immune activity at the periphery of tumor (Figure 7g) in contrast to infiltration into the central tumor (Figure S4c). Because of the detection limits of bioluminescence microscopy, L-012 imaging was only achieved using abdominal window chambers as the gut showed higher L-012 activity compared to skin window chambers, as previously reported [32].

As a further demonstration of the versatility of this imaging strategy, abdominal window chambers were also implanted onto transgenic reporter mice to image the pancreas [32]. An abdominal window chamber was implanted onto the spontaneous murine pancreatic ductal adenocarcinoma (PDAC) model, *LSL-Kras^G12D/+^;LSL-p53^T172H/+^;Pdx1-Cre* (*KPC*), which was bred with *ROSA26-pGAGGs-LSL*-*luciferase* animals, resulting in a spontaneous bioluminescent PDAC animal model (*KPC-Luc*). *KPC-Luc* animals developed spontaneously bioluminescent pancreas tumors around 15–20 weeks of age [38]. Abdominal window chambers were implanted on *KPC-Luc* and *KPC* control (no bioluminescent tumor reporter) animals at 14 weeks of age. Pancreas tumor growth was imaged using FLuc bioluminescence at 2X and 10X (Figure 7h). In *KPC* control animals, L-012 bioluminescence readily detected innate immune infiltrates (Figure S4d). Fluorescence imaging of vasculature and myeloid cells [9,40,41,42,50] was demonstrated following intravenous injection of dextran-FITC (Figure 7h, dextran-FITC and Figure S4d, dextran-FITC). In another example, abdominal window chambers were also implanted on knock-in *p*21 luciferase (*p*21-*FLuc*) transcriptional reporter mice [26] with and without Pan02 pancreatic tumor cells stably expressing the CMV-driven Dendra2 fluorophore (Pan02 Dendra2). Bioluminescence imaging in *p*21-*FLuc* animals now monitored endogenous *p*21 promoter activity [26]. Interestingly, bioluminescence *p*21 activity was reduced in the surrounding microenvironment in tumor-bearing animals compared to healthy animals at both 2X (Figure S4e) and 10X magnification (Figure S4f).

This section may be divided by subheadings. It should provide a concise and precise description of the experimental results, their interpretation, as well as the experimental conclusions that can be drawn.

## 4. Discussion

A variety of custom-built intravital microscopy systems have been reported by expert laboratories [1,2,3,4,5,6,7,40,41,42,53,54,55,56,57,58,59,60,61,62]. Our goal was to expand the utility of the intravital imaging strategy by first starting with an off-the-shelf commercial system, and by modifying this to achieve high quality imaging within the reach of any biology laboratory. This intravital microscopic imaging platform, which combines both bioluminescent and fluorescent genetically encoded reporters, as well as exogenous reporters, provides a powerful multi-plex strategy to study molecular and cellular processes in real-time in intact living systems at single cell resolution. The combination of bioluminescence and fluorescence capacity with intravital microscopy all in a single system provided a first-in-class advance in technology for the high-resolution multi-modal imaging of single cells, signaling dynamics, and real-time spectral unmixing of heterogeneous living systems in vivo. The addition of bioluminescence imaging to a fluorescence microscope was achieved by the addition of a high quality, cooled, back-illuminated CCD camera. These cameras also are often “plug and play” ready, with well-controlled background, and validated linear response, all critical for not only detecting but measuring changes in emitted light. As new detector schemes become integrated into vendor software, further integrations are expected to accommodate EMCCD, ICCD, cooled scientific CMOS and other emergent camera technologies. While minimizing stray external light was readily achieved, significant effort was expended in identifying key internal sources of stray light, many of which may be vendor specific. Nonetheless, careful assessment and strategies to block stray photons can be achieved in low-noise systems.

Intravital bioluminescence microscopy demonstrated the heterogeneous activation of NF-κB within melanoma tumors, which was not captured by a matched macroscopic imaging system [32]. Baseline heterogeneous NF-κB signaling levels within individual cells of a tumor and between individual tumors were confirmed using two different bioluminescence-based NF-κB transcriptional activation reporters at single cell resolution, as well as in two different tumor models (melanoma and pancreas). Real-time changes in NF-κB cell signaling dynamics within individual cells of a tumor were demonstrated using TNFα-induced activation during bioluminescence microscopy in vivo. NF-κB signaling was examined due to the biological significance of this pathway; NF-κB is considered a central coordinator of the immune system and implicated during cancer development [63,64,65,66]. The visualization of bioluminescence micro-structures by microscopy, but not by macro-imaging, was the result of the change in sensitivity, pixel size and effective f stop between the systems. Photon-emitting sources arising from single cells or a cluster of cells that were smaller than the macro-imaging pixel size often were undetectable by macro-imaging due to partial volume effects, which were not a limitation of the higher resolution microscopy system. However, by leveraging the strengths of both systems, multi-modal, multi-spectral whole body (and whole window chamber) imaging [32] could be combined with dynamic molecular signaling and cellular imaging, providing multi-scale information of disease progression in an individual animal. Furthermore, with the high correlation between the widely utilized macro-imaging systems (IVIS) and the 2X objective on the custom microscopy system, global trends could be interrogated and cross-validated on both imaging platforms.

Herein, we also demonstrated that bioluminescent reporters were complimentary to fluorescent reporters. The inclusion of bioluminescent reporters did not affect fluorescence imaging and vice versa, demonstrated by using genetically encoded reporters expressed in both implanted tumor cells and transgenic reporter animals. Photomarking tumor cells using a photoswitchable fluorophore, Dendra2, was also not affected by the presence of bioluminescent reporters, nor did photoconversion affect bioluminescence signals. The inclusion of a photoswitchable fluorophore allowed cells, or areas of interest, to be marked, and changes in signaling dynamics, or cell–cell interactions could be followed for many days throughout disease progression. However, when combining a bioluminescent signaling reporter with a constitutively active photoswitchable fluorophore, if photoconversion occurs, challenges can arise in the normalization of the bioluminescence signal to tumor mass fluorescence due to difficulties in controlling the quantity of photoconversion, the differences in the fluorescence intensity between unphotoswitched and photoswitched signals, and the loss of photon converted signal over time. When using photoconverted fluorescent proteins, the normalization of bioluminescence signaling dynamics to initial bioluminescence may be better suited for quantification purposes when comparing between different animals. The capability to spectrally resolve different luciferases that use the same substrate, as well as bioluminescent reporters that use different substrates, was also achieved on a microscopic level in vivo.

As we have demonstrated, this platform could be used to image, in real time, the interaction between cancer cells and immune cells and could be further extended to other diseases in vivo. With the continued development of genetically encoded imaging reporters, different signaling networks, as well as changes in transcription, translation, protein folding, protein–protein interactions, protein degradation and second messengers could be studied. We demonstrated the spectral resolution of up to four imaging reporters (two bioluminescent and two fluorescent reporters). However, in principle, our current intravital microscopy system has the capability to resolve three bioluminescence and four fluorescence channels. Herein, we demonstrated two different permanent window chamber models—skin and abdomen—but numerous other tissue areas have also been imaged by fluorescence window chamber imaging, including the liver, spleen, small intestine, brain, lung, femur, mammary fat pad, lymphodes, and spine [4,39,53,54,67,68,69,70,71,72,73]; these window chamber models could easily be extended to include in vivo bioluminescence. The use of our intravital microscopy imaging platform provides a strategy to begin to understand how cells communicate as well as changes in signaling networks, both spatially and temporally, in a quantitative manner in a heterogeneous living system at cellular resolution. With the paradoxical dual role that the immune microenvironment plays during cancer progression, where it can restrain or promote cancer progression [12,13,14,15], it is essential to develop strategies to better understand the interaction networks between tumor cells and immune cells to improve disease detection and monitor therapeutic response.

## Data Availability

All data presented within this study are available within the manuscript and the Supplementary Materials.

## Supplementary Materials

The following are available online at https://www.mdpi.com/2073-4409/10/3/499/s1, Figure S1. Quantification of the elimination of stray light sources. Figure S2. Intravital imaging of skin window chamber-bearing animals of tumor NF-κB transcriptional activation. Figure S3. B16F10 Dendra2 photoconversion in skin window chamber-bearing animals. Figure S4. Intravital microscopy of abdominal window chamber animals.
