# Supplementary material for: Multi-Modal Multi-Spectral Intravital Microscopic Imaging of Signaling Dynamics in Real-Time during Tumor–Immune Interactions
Source: Cells. 2021 Feb 26;10(3):499. doi: 10.3390/cells10030499 (PMC7996937; doi:10.3390/cells10030499)
Supplement: Supplementary file 1 [file cells-10-00499-s001.pdf]

cells

Supplemental Materials

Supplemental Figures

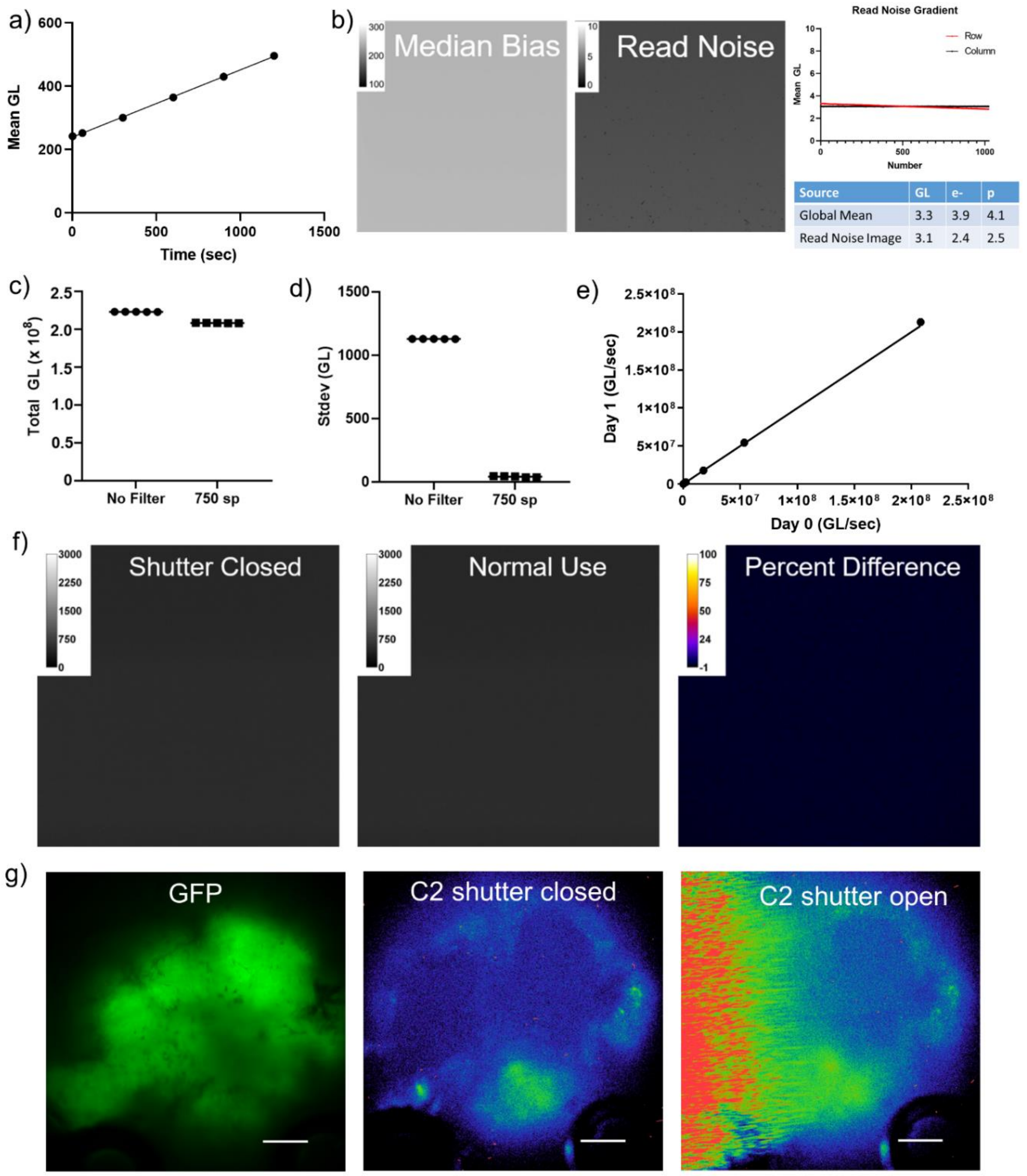

Figure S1. Quantification of the elimination of stray light sources. (**a**) Images were acquired with the camera shutter closed to establish mean dark current. Light quantified as grey levels (GL) vs time (n = 5 images per time) was plotted, yielding a slope of 0.208 mean GL/(sec*pixel), $R^2 = 0.999$; data were expressed as mean ± SEM (error bars fall within the data point). (**b**) Camera characterization of read noise globally and on a per pixel basis with the camera shutter closed; read noise was well controlled (3 GL) with very small but detectable top to bottom and left to right gradients in read noise: 4.9×10^{-4} GL/pixel and 1.810^{-6} GL/pixel, respectively. Quantification of the (**c**) total GL of images and (**d**) standard deviation (Stdev); 2X objective, 20 min acquisition with the 750 nm short pass filter (750 sp)

and without the filter (No Filter). (**e**) Light standard measurements (n = 5) on day 0 and day 1 plotted against each other; linear fit had a slope of 1 indicating excellent day to day test-retest agreement (n = 5 images, data were expressed as mean ± SEM (error bars fall within the data point)). (**f**) Representative stress test images (2X objective, 20 min acquisition, n = 5 images per group) with camera shutter closed and open (normal use); the difference between normal use and shutter closed images was calculated and displayed on a pixel wise basis as percent difference. (**g**) Representative intravital images of skin window chamber-bearing animals of tumor GFP (GFP cube, 1 s exposure) and bioluminescent tumor NF-κB transcriptional activation with the C2 shutter closed and open ((BLI, 20 min acquisition, open filter) at 2X; scale bar represents 1000 μm.

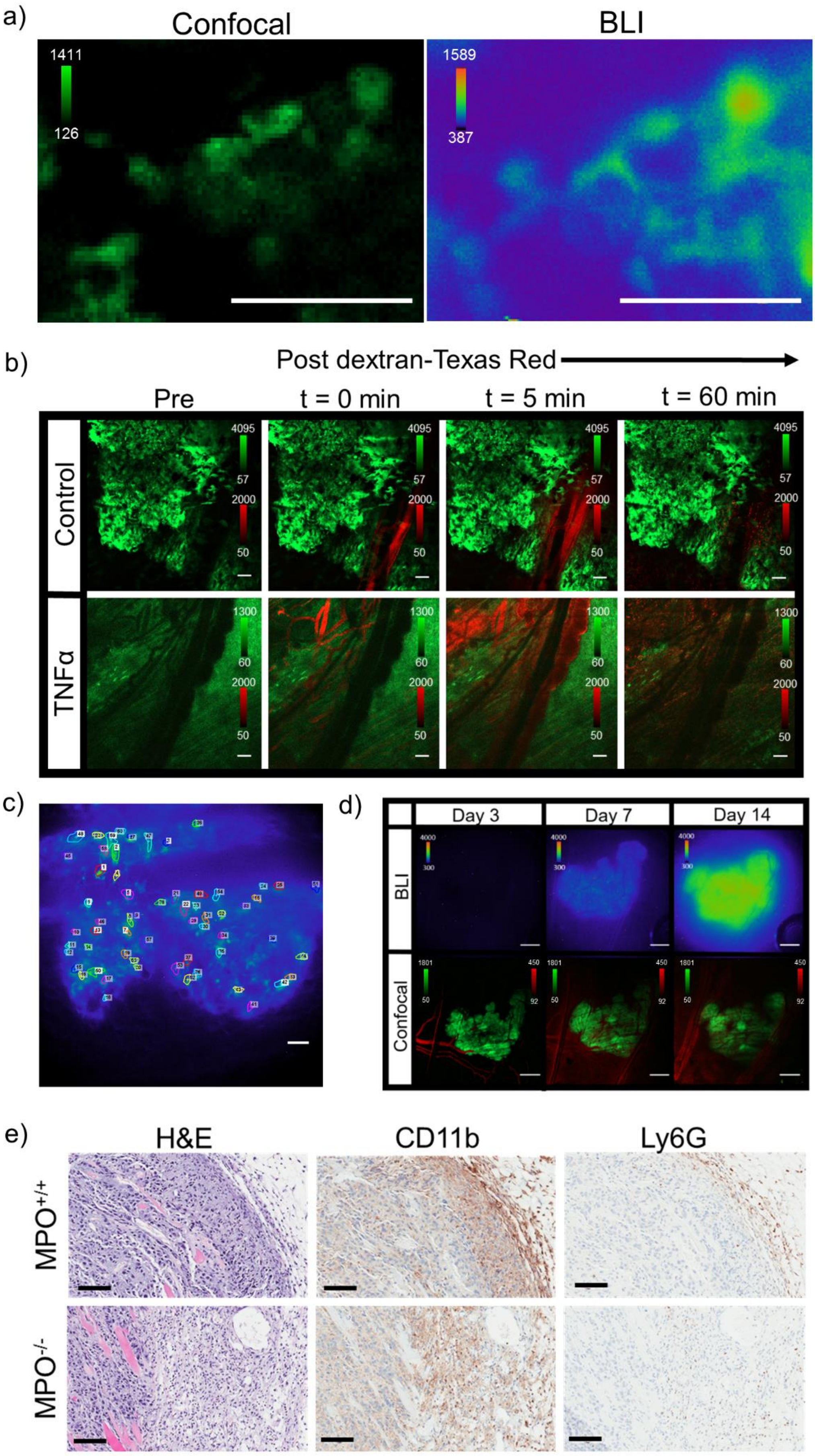

a)
Confocal
BLI
1411
126
1589
387
b)
Post dextran-Texas Red
Pre
t = 0 min
t = 5 min
t = 60 min
Control
TNFα
4095
57
2000
50
1300
60
c)
d)
Day 3
Day 7
Day 14
BLI
Confocal
4000
300
1801
50
450
92
e)
H&E
CD11b
Ly6G
MPO+/+
MPO-/-

Figure S2. Intravital imaging of skin window chamber-bearing animals of tumor NF-κB transcriptional activation. (**a**) Representative intravital image of B16F10 κB-FLuc GFP tumors in C57BL/6 window chamber-bearing animal using confocal imaging of tumor cells (green) and corresponding NF-κB bioluminescence (BLI) image (10X objective, scale bar represents 200 μm). (**b**) Representative time course intravital images of B16F10 κB-FLuc GFP tumors in C57BL/6 window chamber-bearing animal using confocal imaging of tumor (green) and vasculature (red) fluorescence following i.v. injection of dextran-Texas Red alone (control) and with TNFα (10X objective, 4.8 speed, laser lines: 488 nm (GFP, tumor) and 561 nm (dextran-Texas Red, vasculature); scale bar represents 100 μm). (**c**) Representative image of ROIs used to quantify κB-FLuc bioluminescence of single tumor cells and cluster of tumor cells within a bulk tumor at 10X. (**d**) Representative intravital images of C57BL/6 window chamber-bearing animal with B16F10 NF-κB-FLuc Dendra2 tumor growth over time; bioluminescence (BLI, 20 min acquisition, open filter) and Dendra2 epifluorescence (GFP cube, 1 s exposure) at 2X; scale bar represents 1000 μm. (**e**) Representative immunohistology of $MPO^{+/+}$ and $MPO^{-/-}$ window chamber skin flaps at endpoint showing H&E, CD11b and Ly6G staining confirming presence of myeloid cells (20X; scale bar, 100 μm).

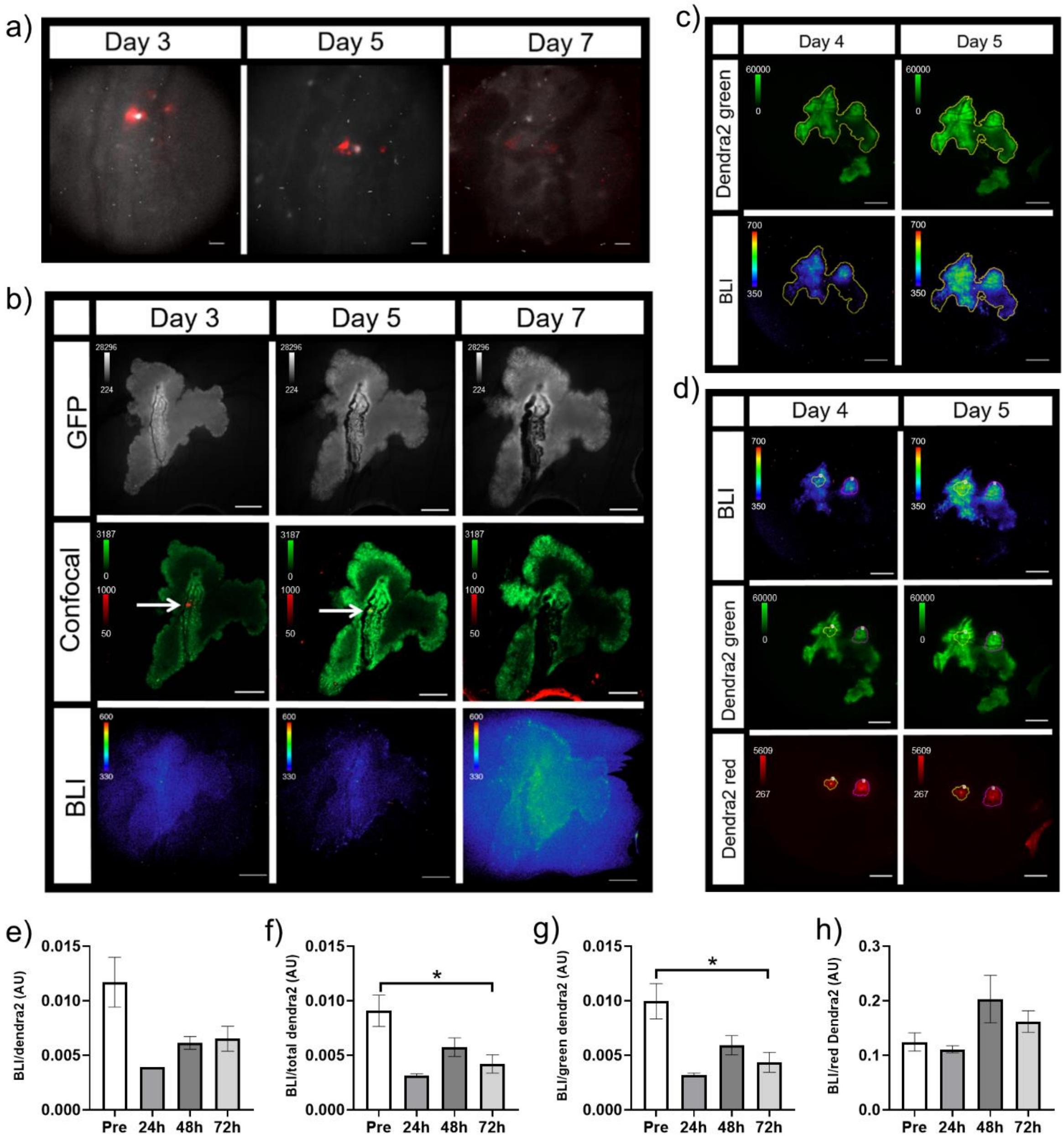

Figure S3. B16F10 Dendra2 photoconversion in skin window chamber-bearing animals. (**a**) Representative intravital images over time of photoswitched Dendra2 (red, confocal image: 4.8 speed, laser line 561 nm) overlaid with corresponding bioluminescent image (BLI, 20 min acquisition, open filter) at 10X; scale bar represents 100 μm. (**b**) Representative intravital image of B16F10 NF-κB-FLuc Dendra2 tumors over time following single cell 405 nm confocal laser photoconversion of Dendra2 epifluorescence (GFP cube, 2 s exposure), merged Dendra2 confocal image (4.8 speed, laser lines: 488 nm (green unphotoconverted Dendra2) and 561 nm (red photoconverted Dendra2)) and NF-κB-FLuc bioluminescence (BLI, 20 min acquisition, open filter) at 2X; white arrow identifies photoswitched cell; scale bar represents 1000 μm. (**c**) Representative image of auto threshold ROIs of whole tumor showing unphotoconverted Dendra2 fluorescence (Dendra2 green) and (**d**) photoconverted areas within the same tumor using photoconverted Dendra2 fluorescence (Dendra2 red). (**e**) Normalization of quantified bioluminescent NF-κB signal using Dendra2 fluorescent intensity of green unphotoconverted Dendra2 from total tumor area. Quantified bioluminescent NF-κB signal normalized to Dendra2 fluorescent intensity of the photoconverted tumor areas using (**f**) sum of unphotoconverted green and photoconverted red Dendra2 fluorescence, (**g**) green unphotoconverted Dendra2 only, and (**h**) red photoconverted Dendra2 only (data are represented as mean ± SEM,

statistical significance calculated using mixed-effects analysis with Dunnett's multiple comparisons * $P < 0.05$).

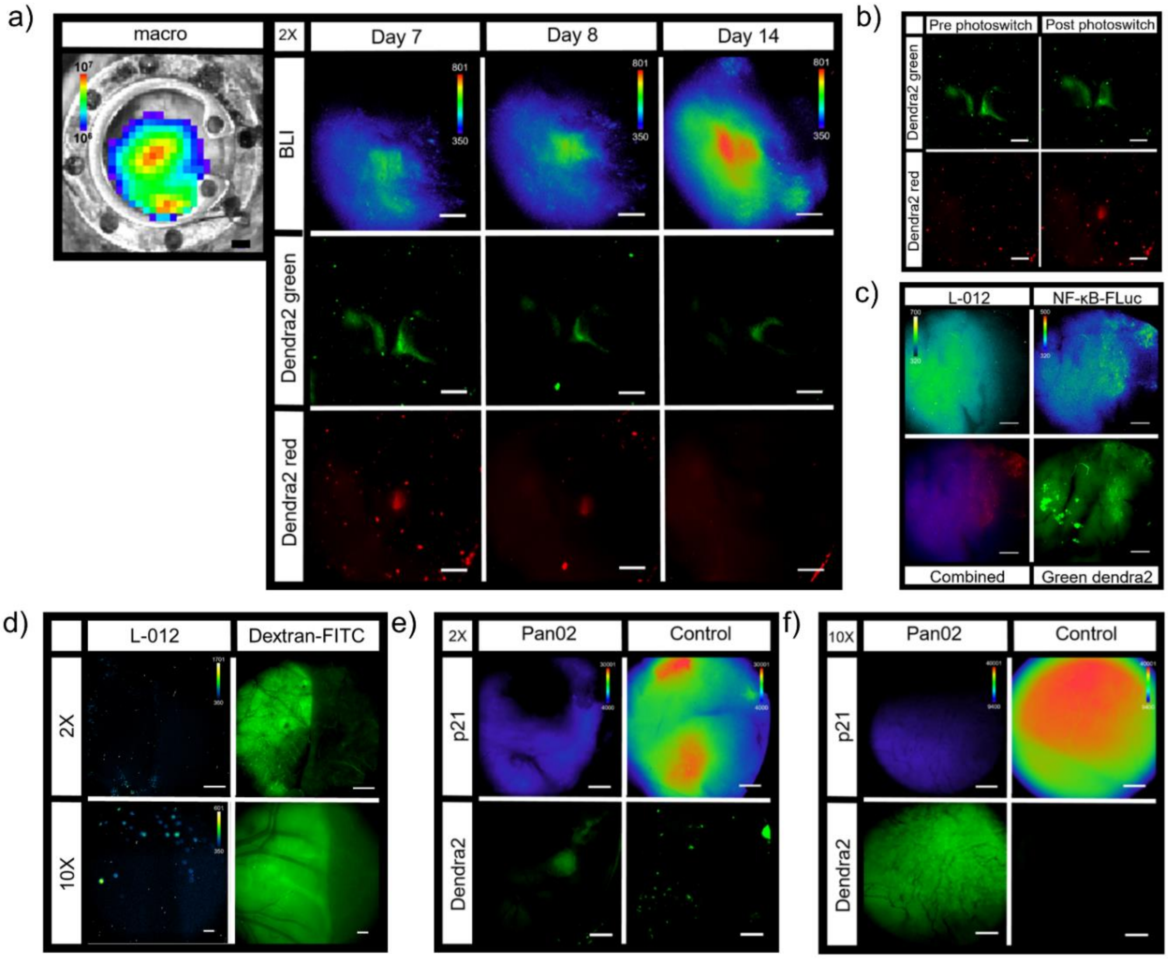

Figure S4. Intravital microscopy of abdominal window chamber animals. (**a**) Representative intravital images of the pancreas using wild type C57BL/6 abdominal window chamber animals with Pan02 NF-κB-FLuc Dendra2 pancreatic tumors. NF-κB-FLuc bioluminescence and Dendra2 fluorescence images of implanted Pan02 pancreatic tumor progression imaged by macro-image (macro) and over time at 2X (BLI, 10 min acquisition, open filter; Dendra2 green, GFP cube, 2 s exposure; Dendra2 red, DsRed cube, 5 s; scale bar represents 1000 μm). (**b**) Representative fluorescent image of Pan02 tumor pre- and post-photoswitching (DAPI cube, 10 min exposure) at 2X objective (Dendra2 green, GFP cube, 2 s exposure; Dendra2 red, DsRed cube, 5 s; scale bar represents 1000 μm). (**c**) Representative intravital images of resolved bioluminescence of L-012 innate immune cell activity and Pan02 NF-κB-FLuc reporters (2X objective, sequential imaging of L-012 (20 min acquisition, open filter) and NF-κB-FLuc (10 min acquisition, open filter)), overlaid image of bioluminescent reporters (combined; pseudocolored), and tumor Dendra2 (Dendra2 green, GFP cube, 2 s exposure); scale bar represents 1000 μm. (**d**) Representative intravital images of the pancreas in control *KPC* transgenic animals implanted with an abdominal window chamber showing L-012 bioluminescence (20 min acquisition, open filter) and vasculature following i.v. injection of dextran-FITC (200,000 MW, 4 mg/mL; 2X, 5 sec exposure; 10X, 1 sec exposure); scale bar represents 1000 μm and 100 μm, respectively, n = 2 animals. (**e**, **f**) Representative intravital images of the pancreas in *p21-Fluc* transgenic reporter animals implanted with an abdominal window chamber and a Pan02 Dendra2 tumor (Pan02) or without a tumor (control) at day 13 post implantation. Reporter *p21* promoter activation is monitored by FLuc bioluminescence following D-luciferin injection and Pan02 tumor mass monitored using Dendra2 fluorescence at (**e**) 2X (*p21* BLI, 5 min acquisition, open filter; Dendra2, GFP cube, 5 sec exposure; scale bar represents 1000 μm) and (f) 10X (*p21* BLI, 10 min acquisition, open filter; Dendra2, GFP cube, 1 sec exposure; scale bar represents 100 μm); n = 2 animals per group.
